# UINMF performs mosaic integration of single-cell multi-omic datasets using nonnegative matrix factorization

**DOI:** 10.1038/s41467-022-28431-4

**Published:** 2022-02-09

**Authors:** April R. Kriebel, Joshua D. Welch

**Affiliations:** 1 Department of Computational Medicine and Bioinformatics, University of Michigan, Ann Arbor, MI USA; 2 Department of Computer Science and Engineering, University of Michigan, Ann Arbor, MI USA

**Keywords:** Data integration, Machine learning

## Abstract

Single-cell genomic technologies provide an unprecedented opportunity to define molecular cell types in a data-driven fashion, but present unique data integration challenges. Many analyses require “mosaic integration”, including both features shared across datasets and features exclusive to a single experiment. Previous computational integration approaches require that the input matrices share the same number of either genes or cells, and thus can use only shared features. To address this limitation, we derive a nonnegative matrix factorization algorithm for integrating single-cell datasets containing both shared and unshared features. The key advance is incorporating an additional metagene matrix that allows unshared features to inform the factorization. We demonstrate that incorporating unshared features significantly improves integration of single-cell RNA-seq, spatial transcriptomic, SNARE-seq, and cross-species datasets. We have incorporated the UINMF algorithm into the open-source LIGER R package (https://github.com/welch-lab/liger).

## Abstract (web summary)

Single-cell genomic technologies present unique data integration challenges. Here the authors introduce an integrative nonnegative matrix factorization algorithm that incorporates features unshared between datasets when performing dataset integrations, improving integration results for spatial transcriptomic, cross-modality, and cross-species data.

## Introduction

Each cell type or state within an organism is distinguished by its gene expression, epigenetic regulation, and spatial location within a tissue. Single-cell sequencing technologies measure each of these features in individual cells, allowing researchers to classify cells in a data-driven manner. Determining what features are common, or different, between each cell type provides researchers insight into the function of the cell. Comparing the profiles of diseased cells with those of healthy cells also reveals disease-related aberrant features. An ideal characterization of a cell type goes beyond analyzing features such as epigenetic and gene expression individually, instead examining their relationships. Jointly examining cellular features holds promise for understanding gene regulatory mechanisms that control cell fates.

Current single-cell sequencing technologies cannot simultaneously measure all relevant aspects of cell state. Recently developed multi-omics technologies are limited to capturing only a couple of the modality measures that encompass cell identity^1^. In particular, emerging techniques such as the Multiome assay from 10X Genomics can measure gene expression and chromatin accessibility from the same cell^2–7^, but do not generally capture methylation or spatial features. Spatial transcriptomics, named Method of the Year 2020 by Nature Methods^8^, encompasses a rapidly growing suite of techniques^9–12^ that interrogate gene expression patterns within intact tissue. However, protocols for spatial measurements of epigenomic state are not widely available.

The different types of features measured by different single-cell technologies create unique computational data integration challenges. Some more recent methods, such as Seurat v4’s Weighted Nearest Neighbor (WNN) algorithm^13^, are designed for datasets containing multiple modalities measured within the same cells, while other approaches focus on integrating modalities from different single cells into a shared latent space. Most existing computational approaches for multi-omic data integration are designed for either vertical or horizontal integration scenarios^14,15^. Vertical integration approaches are useful for datasets measured across a common set of samples or cells. Well-established methods for multi-omic integration of bulk data, such as similarity network fusion and iCluster^16,17^, fall into this category, as well as recent methods for single-cell datasets with multiple modalities per cell such as MOFA+, totalVI, and the Seurat v4 weighted nearest neighbors algorithm^18–20^. Conversely, horizontal integration uses a set of common variables or features to integrate over multiple experiments, typically using shared genes as the anchors for integration. Batch effect correction approaches originally designed for bulk sequencing data (e.g., RUV^21,22^ and ComBat^23^) solve a horizontal integration problem. Similarly, dataset alignment algorithms developed for single-cell data, such as Seurat v3, Harmony, and our previous method LIGER^18,24–26^ also rely on shared features and can thus be considered horizontal integration techniques.

A recent review of single-cell computational integration approaches identified a third category of approaches as those for diagonal integration, in which neither features nor cells are assumed to be shared across datasets^15^. In this review, Argelauget et al. also identified a type of problem that they call mosaic integration–in which only some features or cells are shared across datasets–and for which they did not identify any existing approaches. Our work here aims to address some of the challenges of mosaic integration.

LIGER, Seurat v3, and Harmony are effective tools for a number of single-cell integration tasks, and were chosen as the best methods in a recent systematic comparison of 14 computational methods^27^. However, these three approaches are all constrained to integrate across features shared between datasets, and require that the input matrices all contain a common set of genes or features that are measured in all datasets. Thus, these methods cannot incorporate features unique to one or more datasets, such as intergenic epigenomic information.

Restricting single-cell integration analyses to features shared across all datasets is problematic because it often necessitates discarding pertinent information. For instance, scRNA-seq measures transcriptome-wide gene expression within individual cells, but spatial transcriptomic protocols often measure only a chosen subset of all genes. Yet for many applications, we want to integrate scRNA-seq and spatial transcriptomic datasets, which have neither the same number of features (genes) nor the same number of observations (cells). By integrating the datasets using only shared features, we fail to capitalize on the higher resolution provided by the scRNA-seq modality. When integrating cross-species datasets, the integration is restricted to orthologous genes, disregarding all genes without unambiguous one-to-one relationships between species. Likewise, when integrating single-cell epigenomic data with single-cell transcriptomic data, horizontal integration approaches do not take into account the important epigenomic features from intergenic regulatory elements. As a final example, existing methods do not provide a way to leverage paired epigenomic information when integrating data types such as SNARE-seq^2,5^ or 10X Multiome with single-cell or spatial transcriptomic datasets. Such integration analyses do not fit neatly into either the horizontal or vertical integration paradigm, requiring the development of different methods.

The critical need to include unshared features in single-cell integration analyses motivated us to extend our previous approach. We developed UINMF, a nonnegative matrix factorization algorithm that allows the inclusion of both shared and unshared features. UINMF can integrate data matrices with neither the same number of features (e.g., genes, peaks, or bins) nor the same number of observations (cells). Furthermore, UINMF does not require any information about the correspondence between shared and unshared features, such as links between genes and intergenic peaks. By incorporating unshared features, UINMF fully utilizes the available data when estimating metagenes and matrix factors, significantly improving sensitivity for resolving cellular distinctions. Note that UINMF is not designed to jointly leverage multiple modalities from the same cell as in Seurat v4 or MOFA; rather, the approach solves a problem more like the Seurat v3 anchors approach.

## Results

### Integrative nonnegative matrix factorization algorithm for partially overlapping feature sets

**Fig. 1:**
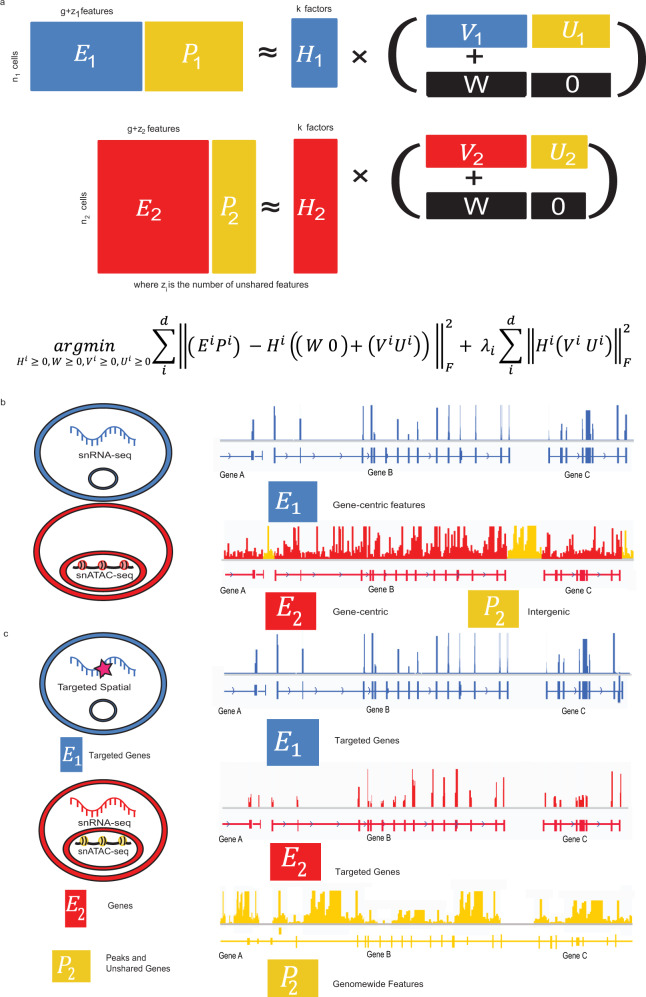
Overview of the UINMF algorithm for integrating single-cell datasets with partially overlapping features. **a** Schematic representation of the matrix factorization strategy (top) and optimization problem formulation (bottom). The addition of a factor matrix *U*^i^ allows for unshared features to be utilized in joint matrix factorization. Each dataset (*E*^*i*^) is decomposed into shared metagenes (*W*), dataset-specific metagenes constructed from shared features (*V*^*i*^), unshared metagenes (*U*^*i*^), and cell factor loadings (*H*^*i*^). The incorporation of the *U* matrix allows unshared features that occur in only one dataset to inform the resulting integration. **b** UINMF can integrate data types such as scRNA-seq and snATAC-seq using both gene-centric features and intergenic information. **c** UINMF can integrate targeted spatial transcriptomic data with simultaneous single-cell RNA and chromatin accessibility measurements using both unshared epigenomic information and unshared genes.

The key innovation of UINMF is the introduction of an unshared metagene matrix *U* to the iNMF objective function, incorporating features that belong to only one, or a subset, of the datasets when estimating metagenes and cell factor loadings. Previously, dataset integration using the iNMF algorithm operated on features common to all datasets^25^. Each dataset (*E*^*i*^) was decomposed into dataset-specific metagenes (*V*^*i*^), shared metagenes (*W*), and cell factor loadings (*H*^*i*^), and the optimization problem was solved iteratively. By including an unshared metagene matrix (*U*^*i*^), we provide the capability to include unshared features during each iteration of the optimization algorithm (Fig. 1a). Intuitively, the shared features allow for the identification of corresponding cells across datasets, while the unshared features incorporated by *U*^*i*^ simultaneously allow for  clearer distinction among the cells within each dataset.

The unshared feature matrix can include extra genes, intergenic features, non-orthologous genes, or any other data type that is measured in one of the datasets. Importantly, UINMF makes no assumptions about the relationship between the unshared features and the shared features; for example, no prior knowledge about linkages between intergenic peaks and genes is required. Instead, such covariance among features is learned during the optimization process, as both shared and unshared features contribute to the reconstruction of the original data through the inferred latent factors. These properties allow for the use of the UINMF algorithm across diverse contexts. For example, *U*^*i*^ can incorporate intergenic information when integrating single-cell transcriptomic and epigenetic datasets (Fig. 1b). When analyzing spatial transcriptomic datasets measuring only a few targeted genes, *U*^*i*^ can be used for genes that are not in the targeted panel but are measured in transcriptome-wide scRNA-seq data. With the advent of multi-omic datasets, the flexible nature of *U*^*i*^ is a particular advantage. We can jointly integrate single-modality data with multimodal data, using all of the features present in the multimodal dataset when performing the integration. In this scenario, the *U*^*i*^ matrix can be used for the unshared multimodal data type, such as chromatin accessibility, when integrating SNARE-seq data with spatial transcriptomic or scRNA data (Fig. 1c). Another application of the unshared matrix is the ability to include non-orthologous genes into cross-species analyses, leveraging species-specific genes in dataset integration. We demonstrate the functionality of the UINMF algorithm in each of these four possible scenarios, and anticipate that the approach will prove useful for a wide variety of future applications.

The UINMF optimization algorithm has a reduced computational complexity per iteration compared to our previous iNMF algorithm^25^ on a dataset of the same size (see Methods). Furthermore, the addition of the unshared features in each iteration of the matrix decomposition does not impose a prohibitive increase in time or memory usage in practice. Rather, UINMF demonstrates a moderate increase in time and memory usage due to the unshared features while providing significant benefits compared to algorithms incapable of incorporating unshared features (Supplemental Fig. 1).

UINMF, as well as iNMF, requires random initializations, and is nondeterministic in nature. Therefore, unless otherwise noted, we perform UINMF and iNMF with multiple initializations, and use the model of best fit for each respective algorithm. In practice, the use of a single initialization, set with a random seed, is typically sufficient.

As an initial test of UINMF, we investigated how differing numbers of shared and unshared features affect integration quality. To do this, we integrated two single-cell transcriptomic datasets taken from the mouse Primary Motor Area (MOp)^13^, sequenced with two different protocols: SMART-seq and 10X Genomics. Using a constant number of total genes (2500), we progressively decreased the number of shared genes from 500 to 100. We then performed two UINMF integrations, one using the SMART-seq data as the source for the unshared features, and the other using the 10X dataset as the source for the unshared features. We calculated the ARI and purity scores for each resulting integration (Supplemental Fig. 2).

Our results indicate that using unshared genes from the SMART-seq data, known to be higher quality than the 10X data, consistently increases the quality of dataset integration, even with a continued reduction in the number of shared genes. The 10X data, while initially improving dataset integration, is more dramatically impacted by the reduction of shared features. Nevertheless, the benefit of using UINMF to include additional features in dataset integrations is clear, with the amount of benefit derived correlated with the quality of the dataset from which the unshared features are being chosen.

### Including intergenic peak information improves integration of scRNA and snATAC datasets

**Fig. 2:**
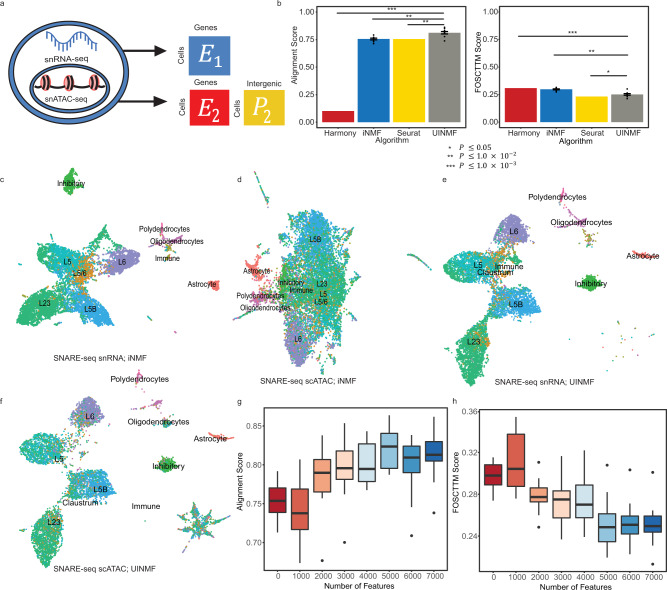
Addition of intergenic peak information improves integration of RNA and ATAC datasets. **a** Schematic illustrating how the UINMF algorithm incorporates intergenic peaks when separately integrating the RNA and ATAC measurements from a SNARE-seq dataset. We treat each data type as if it came from an independent source, and perform an integration using regular iNMF and our proposed UINMF method, which incorporates intergenic peaks. **b** Average alignment and FOSCTTM (Fraction of Samples Closer Than True Match) scores for iNMF, Seurat v3, Harmony, and UINMF. iNMF and UINMF are both initialized 5 different times over ten random seeds, with UINMF including an additional 7,000 intergenic features into the analysis. For nondeterministic algorithms, data are presented as mean values +/− SEM. To compare algorithm performance, we used a paired, one-sided Wilcoxon test to compare UINMF’s alignment and FOSCTTM scores to iNMF (*P* = 1.953 × 10^−3^, *P* = 1.953 × 10^−3^), Seurat (*P* = 1.953 × 10^−3^, *P* = 0.01855), and Harmony (*P* = 9.766 × 10^−4^, *P* = 9.766 × 10^−4^), with Seurat exhibiting a significantly lower FOSCTTM score. For each algorithm, we compare 10 pairs of data points (*n* = 20). We factorize and cluster the cells using their RNA transcripts (**c**) and chromatin accessibility measures (**d**) separately. After integration, we use the known cell correspondences to separately plot the gene expression (**e**) and chromatin accessibility datasets (**f**) from SNARE-seq, colored by the same cell type labels. We assess the contribution of information contained within the intergenic peaks by assessing the alignment (**g**) and FOSCTTM (**h**) scores across a range of included peaks, from 0 unshared features (iNMF) to 7000 unshared intergenic bins, adding 1000 unshared features to each analysis. The bold line indicates the median data value, and the boundaries of each box are defined by the first and third data quartiles (25 and 75%, respectively). The upper (lower) whiskers extend from to highest (lowest) point within 1.5 of the interquartile range. Outliers beyond the whiskers are plotted as points. We calculate FOSCTTM and alignment scores for ten random seeds for each number of unshared features (*n* = 10).

We first investigated how the inclusion of additional features might impact the integration of scRNA and snATAC datasets. In our previous work, we summed ATAC reads that fell within a gene to provide gene-centric ATAC profiles, then used these shared features for integration, neglecting intergenic information^13^. In contrast, UINMF uses the unshared feature matrix to incorporate the ATAC reads present between genes–intergenic peaks–when estimating the metagenes (Fig. 2a). Single-nucleus ATAC data is extremely sparse, with only 1–10% of peaks detected per cell, compared to the 10–45% of genes captured per cell in scRNA sequencing methods^28^. Including the intergenic snATAC data allows more of the detected regions to be used from each cell, a distinct advantage in such sparse datasets. Additionally, the intergenic chromatin peaks provide information about the chromatin state of important *cis*-regulatory elements, such as promoters and enhancers. We hypothesized that the inclusion of this additional information would better resolve molecular distinctions among cells when integrating single-cell transcriptomic and epigenomic datasets.

To quantify how leveraging intergenic features improves dataset integration, we analyzed a SNARE-seq dataset^2^, which provides gene expression and chromatin accessibility information from the same barcoded cell. Because the RNA and ATAC information is measured within the same single cells, the joint profiles provide ground truth cell correspondence information for assessing integration performance. The RNA and ATAC profiles can be preprocessed and integrated as if they come from separate datasets. Subsequently, the success of the integration can be measured by how closely the ATAC and RNA profiles for the same cell are aligned.

We evaluated the quality of SNARE-seq integrations using the Fraction of Samples Closer Than the True Match (FOSCTTM)^3^ metric. The FOSCTTM metric assesses how closely the RNA barcoded cell is placed to its corresponding ATAC barcode in the latent space. Lower FOSCTTM scores are better, indicating that the RNA and ATAC profiles from the same cells have been correctly placed near each other. We also calculated an alignment metric; because the RNA and ATAC datasets come from identical cells, perfect alignment is theoretically achievable and thus the ideal performance is an alignment score of 1.

We assessed the benefit of incorporating intergenic peaks by comparing the UINMF algorithm with our previously published iNMF algorithm^25^, which uses only gene-centric features. Over multiple random initializations, iNMF obtained an average FOSCTTM score of 0.2977 and an average alignment score of 0.756 (Fig. 2b).

In contrast, UINMF achieved a significantly lower average FOSCTTM score of 0.251 (*P* = 1.953 × 10^−3^, paired one-sided Wilcoxon test), as well as a significantly higher average alignment score of 0.812 (*P* = 1.953 × 10^−3^, paired one-sided Wilcoxon test). These findings indicate that incorporating unshared features from the chromatin accessibility data improves the integration of scRNA and snATAC datasets.

We also compared UINMF with Seurat v3 and Harmony. UINMF significantly outperforms Harmony in both alignment (*P* = 9.766 × 10^−4^, paired one-sided Wilcoxon test) and FOSCTTM score (*P* = 9.766 × 10^−4^, paired one-sided Wilcoxon test). Compared to Seurat v3, UINMF had a significantly improved alignment score (*P* = 1.953 × 10^−3^, paired one-sided Wilcoxon test), however, Seurat v3 did have a significantly better FOSCTTM score than UINMF (*P* = 0.01855, paired one-sided Wilcoxon test).

We also confirmed that the RNA and ATAC profiles were mapped to similar cell types. To do this, we manually annotated the cell type labels using marker genes from the scRNA data only. Before integration, the clusters separated much more clearly from gene expression data alone than from chromatin accessibility data alone (Fig. 2c, d). After UINMF integration, the cluster labels aligned well across datasets (Fig. 2e, f), indicating that UINMF identified corresponding cell types even though the single-cell correspondence information was not used by the algorithm. In summary, including intergenic chromatin accessibility information in the integration of scRNA and snATAC data significantly improved the integration results.

To examine how the number of intergenic peaks used affected the resulting integration, we assessed the FOSCTTM and alignment scores for increasing numbers of unshared features. We began by performing the analysis with 0 unshared features (iNMF), taking the best of 5 initializations over 10 seeds, and calculating the alignment and FOSCTTM scores. We then added the 1,000 most variable intergenic bins, and repeated the analysis and metric calculations. Iteratively, we added the next 1000 top variable intergenic bins until we reached 7000 unshared features. As the number of variable intergenic bins used increases, the alignment (Fig. 2g) and FOSCTTM (Fig. 2h) scores show a corresponding stepwise improvement, indicating that each additional set of intergenic bins improves the overall integration. Moreover, the increasingly refined metrics of integration suggest that there is a substantial contribution of information even in the last 2000 variable bins added, as integrating with 7000 intergenic bins has less variance in metric scores than integrating with 5000 intergenic bins.

### Leveraging additional genes improves integration with targeted spatial transcriptomic technologies

We expect the UINMF algorithm to be especially effective for integrating targeted spatial transcriptomic datasets, as the number of genes measured in such datasets is often limited. Integrating such datasets with scRNA-seq provides the opportunity to pair transcriptome-wide profiles from dissociated cells with spatial transcriptomic data. This mitigates the loss of sensitivity for distinguishing cell types, while mapping cell types to their spatial positions within a tissue.

To explore the utility of the UINMF algorithm for integrating targeted spatial transcriptomics and scRNA-seq datasets, we analyzed STARmap data^10^ and scRNA-seq data^29^. We used a STARmap dataset that contains spatial position and transcription level for 28 genes across 31,294 cells within a 3D block of tissue from the mouse frontal cortex. To our knowledge, this dataset is unique in that it is the only dataset of spatially resolved gene expression for multiple genes within a 3D tissue block.

**Fig. 3:**
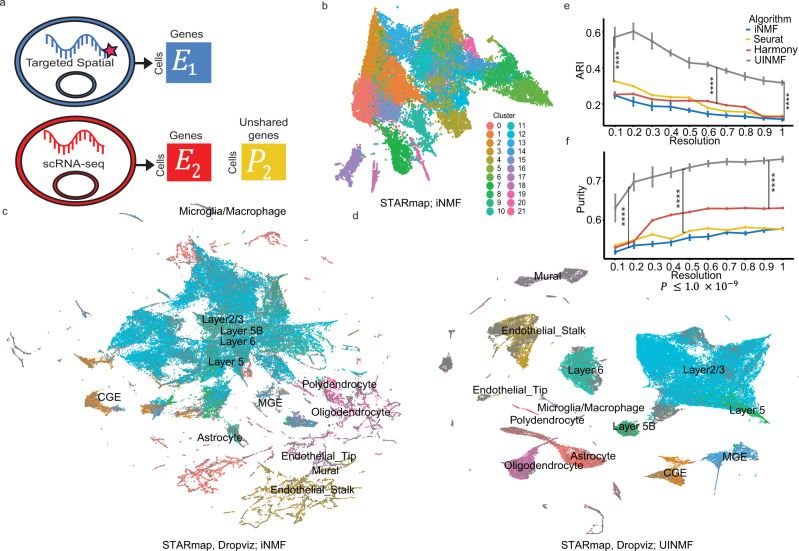
Incorporating additional genes improves integration with STARmap data. **a** Schematic of UINMF integration of the spatial transcriptomic data with the scRNA-seq data, in which *U* incorporates unshared genes that are captured in scRNA-seq but not in the targeted STARmap data. **b** UMAP of STARmap data alone. **c** UMAP of STARmap and scRNA integration performed with iNMF using only shared genes. **d** UMAP of UINMF integration, which incorporates both shared and unshared genes. Both (**c**) and (**d**) are annotated using the original scRNA-seq labels and clusterings derived from either the iNMF (**c**) or UINMF (**d**) algorithm. We compared the adjusted rand index (**e**) and purity (**f**) metrics from UINMF with iNMF (*P* = 3.895 × 10^−10^, *P* = 3.895 × 10^−10^), Seurat v3 (*P* = 3.895 × 10^−10^, *P* = 3.895 × 10^−10^), and Harmony (*P* = 3.895 × 10^−10^, *P* = 3.895 × 10^−10^) for a range of clustering resolutions, using a paired, one-sided Wilcoxon test (*n* = 100 values for each test, with each sample having five values across 10 resolutions). For nondeterministic algorithms, data are presented as mean values +/− SEM.

Even though the 28 genes are selected to distinguish among cell types, the STARmap data fails to clearly separate cortical cell types after matrix decomposition (Fig. 3b). Therefore, we integrate the spatial transcriptomics data with scRNA sequencing data from Saunders et al.^29^ and perform Louvain community detection. We annotate the scRNA-seq cells using their previously published cellular annotations^29^. We observe improved cluster resolution using iNMF with only shared genes to integrate the STARmap dataset with the scRNA-seq dataset (Fig. 3c), but some distinct cell types are mixed together, while others are arbitrarily split. The original cluster labels of the scRNA-seq data are not very well-preserved in the resulting clusters. For example, no distinct boundaries are apparent between the Layer 6, Layer 5, and Layer 5B excitatory neurons. The mural cells are likewise ill-defined. Using UINMF to incorporate 2775 more genes into the integration allows the metagenes to be estimated from a broader array of genes. Consequently, the addition of these unshared features results in dramatically clearer clusters that much better reflect the ground truth labels (Fig. 3d). The distinction between the excitatory neuron subtypes become clear, and a defined population of mural cells also becomes distinguishable. Thus, using the unshared features, it is possible to identify cell types that would not be otherwise distinguishable.

To quantify the advantage of the UINMF method, we calculated Adjusted Rand Index (ARI) and purity metrics for multiple initializations of the UINMF and iNMF algorithms across a range of different Louvain resolution parameters (Fig. 3e, f). The UINMF algorithm achieves significantly (*P* = 3.895 × 10^−10^), paired one-sided Wilcoxon test) higher ARI and cluster purity compared to the iNMF algorithm. UINMF also significantly outperforms both Seurat v3 and Harmony in both ARI and purity metrics (*P* = 3.895 × 10^−10^, paired one-sided Wilcoxon test). In short, the addition of the unshared genes significantly improves the integration of STARmap and single-cell RNA-seq data.

We next examined the contribution of each part of the matrix decomposition (*W*, *V*^1^, *V*^2^, and *U*) for each factor (methods). Both *U* and *V* are regularized in the UINMF objective to encourage the reconstruction to come primarily from the shared factors. Consistent with this regularization, we found that *W*, the shared metagenes, contributed the most to the matrix reconstruction, and the dataset-specific *V*^1^ and *V*^2^ contributed the least (Supplemental Fig. 3a). Intuitively, this allows the shared features to compose the largest scaffold of integration, while the dataset-specific metagenes absorb technical artifacts and biological differences (which should have smaller effects than the shared signals). Interestingly, *U*’s contributions to the matrix reconstruction are generally larger than the contributions from *V*, though still much smaller than those from *W*.

To investigate whether the unshared features *U* highlight any cell-type-specific effects, we ranked the contribution of *U* to each factor, and selected the ten highest factors. We then annotated each factor based on the cell type on which it had the highest loading. The *U* factors with the 10 highest contributions to the reconstructed matrix were primarily non-neuronal (Supplemental Fig. 3b). We suspect that this may be because the selected genes measured by STARmap were primarily marker genes for neuronal cell types. Thus, the inclusion of unshared genes may provide the most benefit for the non-neurons.

**Fig. 4:**
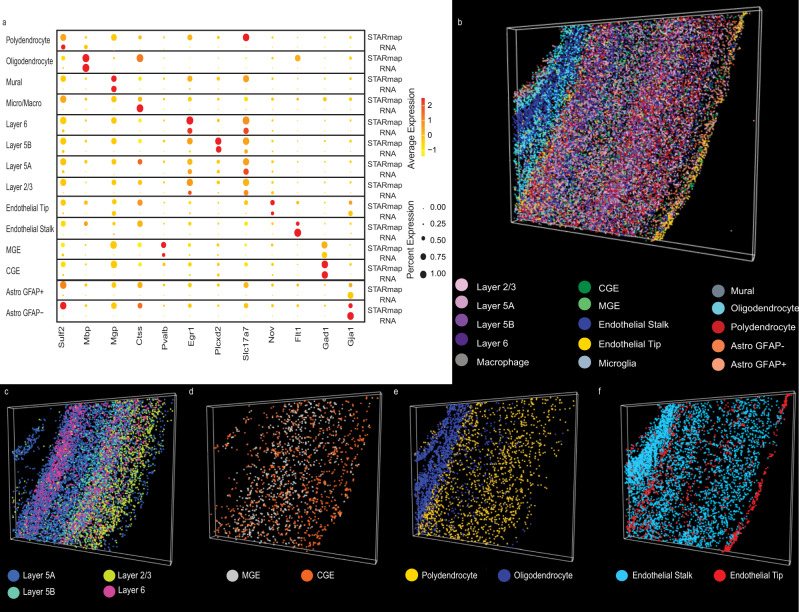
Incorporating additional genes locates fine cellular subtypes within 3D spatial volume. **a** Dot plot showing marker gene expression in STARmap and scRNA datasets for each joint cluster. The datasets have similar marker expression, indicating that they are well aligned. Plots of three-dimensional spatial locations for different classes of cells colored by cell type: (**b**) all cell types; (**c**) excitatory neurons; (**d**) inhibitory neurons; (**e**) polydendrocytes and oligodendrocytes; and (**f**) endothelial cells.

To verify that corresponding cell types were clustered together across technologies, we examined the expression of several key marker genes for each labeled cell type by dataset (Fig. 4a). Generally, marker genes highly elevated in the cell types of the STARmap dataset are also highly elevated in the corresponding scRNA-seq cell type, indicating that the metagene definitions reflect biological distinctions significant for both datasets. This reinforces that the cell clusterings are not reflective of technical artifacts specific to an individual dataset, nor are they formed overwhelmingly by a single dataset. Rather, the clusters reflect divisions significant to both datasets jointly. Additionally, as we previously noted^25^, there is evidence that the STARmap gene capture is somewhat non-specific compared to scRNA-seq for some genes, such as *Sulf2* and *Mgp*.

A key advantage of refining cell types in the STARmap dataset is the ability to use these cell labels within a 3D tissue sample, providing greater insight about how transcriptomic profiles are arranged in vivo. Consequently, we used the derived cell type labels from the UINMF algorithm within the context of 3D space, allowing us to assess their validity on the basis of concordance with known tissue architecture (Fig. 4b). This analysis confirms that our cell type annotations accord well with the known structure of the cortex, such as the clear laminar arrangement of excitatory neurons (Fig. 4c). It has also been previously established that MGE interneurons originate from the more rostral region of the brain, and the CGE neuron center lies caudal to the MGE center^30^. Likewise, the MGE and CGE determined cell types establish a gradient such that the CGE interneurons increase in more cranial regions of the cortex slice (Fig. 4d). The region of white matter that lies beneath the cortex, composed primarily of oligodendrocyte cells, is also identifiable (Fig. 4e). Playing a significant role in supporting the brain’s vascular systems^31^, endothelial cells compose portions of the blood-brain barrier, and the UINMF results indicate that endothelial tip cells are located near the outer surface of the brain (Fig. 4f).

Additionally, we highlight one of the advantages of integrating the STARmap data with scRNA-seq data by imputing values for genes not originally measured in the STARmap assay, e.g., the *Trf* and *Dcn* genes. *Trf* is a recognized marker gene of oligodendrocytes^32^, and *Dcn* has previously been found in three types of vascular leptomeningeal cells (VLMCs)^33^, which are known to comprise vascular structures. Using KNN imputation, we are able to visualize the imputed gene expression within the 3D volume (Supplemental Fig. 4). The expression of *Trf* is primarily confined to the white matter, a region known to be oligodendrocyte-rich, and elevated *Dcn* expression values are visible at the blood-brain barrier.

**Fig. 5:**
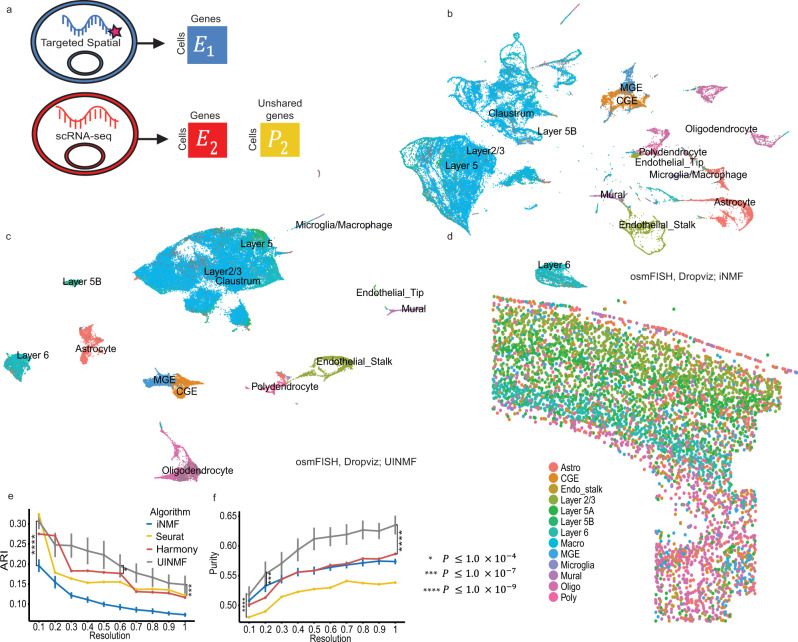
Incorporating additional genes improves integration with osmFISH data. **a** Schematic of data matrices from osmFISH and scRNA-seq. The osmFISH dataset measures only 33 genes, while the scRNA-seq dataset has many unshared genes that are incorporated during UINMF integration. **b** UMAP plot of osmFISH and scRNA integration with iNMF using only shared genes. **c** UMAP using UINMF to incorporate an additional 2,000 genes. Both (**b**) and (**c**) are annotated using the original scRNA-seq labels and either the iNMF (**b**) or UINMF (**c**) derived clusterings. **d** The spatial arrangement of cell types matches the known tissue structure of the cortex (**e**–**f**). ARI (**e**) and purity (**f**) metrics for iNMF (*P* < 2.2 × 10^−16^, *P* = 7.078 × 10^−8^), UINMF, Seurat v3 (*P* = 2.505 × 10^−9^, *P* < 2.2 × 10^−16^), and Harmony (*P* = 2.982 × 10^−5^, *P* = 1.744 × 10^−15^). Statistical significance was evaluated using a paired, one-sided Wilcoxon test (*n* = 200 values for each test, with each sample having 10 values across 10 resolutions). For nondeterministic algorithms, data are presented as mean values +/− SEM.

We further demonstrate the advantages of UINMF for spatial transcriptomic datasets by integrating an osmFISH dataset (5185 cells and 33 genes)^12^ from the mouse somatosensory cortex with the DROPviz scRNA-seq dataset from the frontal cortex (70,020 cells). Using iNMF to integrate the osmFISH and DROPviz datasets utilizes the 33 genes measured in the osmFISH protocol as the shared features (Fig. 5a). The iNMF integration successfully aligns the two data types, but, similar to the STARmap analysis, leads to both mixing and oversplitting of cell types. The intermixing of cell types, especially the excitatory neurons, fails to resolve a distinct cluster for the Layer 5B Excitatory neurons (Fig. 5b). Using UINMF to incorporate an additional 2000 variable genes from the scRNA-seq dataset allows the metagenes to be estimated from many more features, resulting in much more clearly resolved clusters (Fig. 5c). A distinct cluster of Layer 5B Excitatory neurons can now be distinguished. Including additional features into the integration not only categorizes broad cell types more effectively, it also allows for the identification of more minute subclasses of cells.

As with the STARmap data, we then plotted the derived UINMF labels within their corresponding spatial context (Fig. 5d). The excitatory cells again show a clear laminar arrangement, with layer 6 excitatory neurons forming the innermost layer of the cortex, and layer 5 and layer 2/3 neurons above. Interestingly, layer one contains a number of cells identified as astrocytes (Fig. 5d), a finding that has previously been observed experimentally and which has been proposed as evidence for the interaction of glial cells in neuronal signaling^34^. The white matter region, located inferior to layer 6 and known to be composed of oligodendrocytes and polydendrocytes^35,36^, is likewise observable. Lastly, the presence of the caudoputamen and internal capsula region can be identified by the small grouping of inhibitory neurons lateral to the white matter^12^.

To quantify the advantage of using UINMF, we measured the ARI and purity scores for both UINMF and iNMF over multiple initializations and multiple clustering resolutions (Fig. 5e, f). UINMF performed significantly better in terms of both ARI (P < 2.2 × 10^−16^, paired one-sided Wilcoxon test) and purity (*P* = 7.078 × 10^−8^, paired one-sided Wilcoxon test) metrics across the whole range of clustering resolutions. A similar improvement was observed when comparing the UINMF performance to Seurat v3 and Harmony. Note that only a single value is shown for Seurat v3 and Harmony at each resolution because these algorithms are deterministic. UINMF performed significantly better than Seurat v3 and Harmony in both ARI (*P* = 2.505 × 10^−9^, *P* = 2.982 × 10^−5^) and purity (*P* < 2.2 × 10^−16^, *P* = 1.744 × 10^−15^) metrics.

### UINMF improves integration of multimodal and spatial transcriptomic datasets

Single-cell multimodal technologies measure epigenomic and transcriptomic profiles from the same cell, providing an exciting opportunity to define cell types from multiple molecular modalities. However, many applications require integrating such multimodal measurements with single-modality datasets. In such applications, the ability of UINMF to incorporate unshared features allows us to capitalize on the multimodal information, rather than using only shared features.

To demonstrate the advantages of such an approach, we used UINMF to integrate STARmap and SNARE-seq data. The STARmap dataset provides gene expression data for 2522 cells across 1020 genes while preserving the 2D spatial coordinates for each cell. The SNARE-seq dataset (10,309 cells) provides simultaneous chromatin accessibility and gene expression levels from the same barcoded cells.

**Fig. 6:**
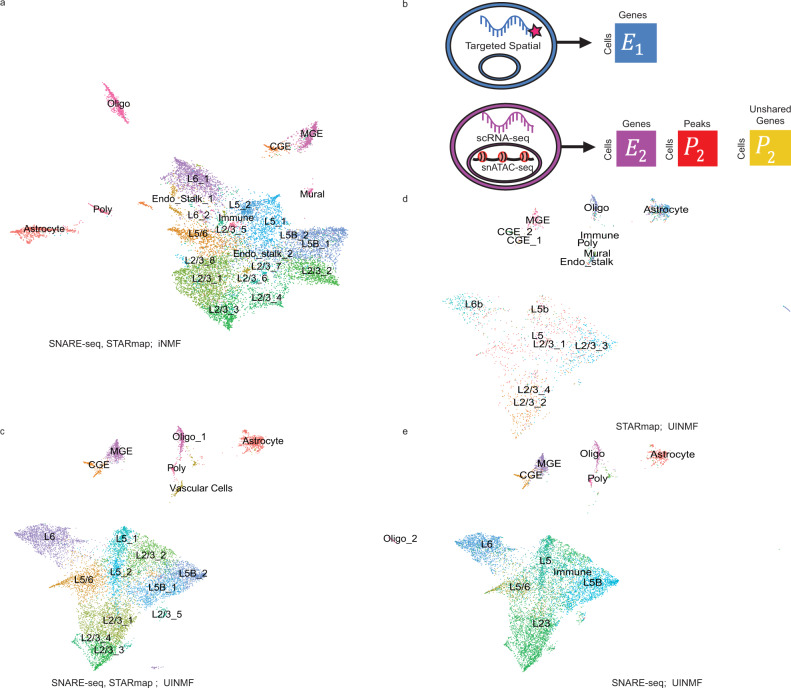
Incorporating unshared chromatin and gene features to integrate spatial transcriptomic and multimodal data. **a** UMAP for iNMF integration of STARmap spatial transcriptomic data and SNARE-seq RNA data only, annotated by jointly examining the SNARE-seq and STARmap labels. **b** Schematic of how unshared gene and chromatin accessibility data is incorporated into the integration analysis of STARmap and SNARE-seq using UINMF. **c** The integration is improved significantly by the inclusion of ATAC gene-centric features in the U matrix, annotated by jointly examining the SNARE-seq and STARmap labels. **d**–**e** The original cell type labels of STARmap cells (**d**) and SNARE-seq cells (**e**) show clear correspondence after UINMF integration.

We first integrated the STARmap data and SNARE-seq gene expression measurements only by performing iNMF on the 944 genes shared between the datasets, omitting the unshared genes and completely neglecting the chromatin information. We annotated the cells by using the original annotations from both datasets to jointly define the resulting clusters (Fig. 6a). When integrating the datasets with UINMF, we were able to add an additional 2688 highly variable genes present in the SNARE-seq dataset when calculating the metagene loadings. Because the SNARE-seq data is multiomic, we also incorporated the available chromatin accessibility information by including the top 1431 variable chromatin accessibility features within the *U* matrix (Fig. 6b). Thus, the UINMF integration incorporated a total of 4119 features not measured in the STARmap dataset (Fig. 6c).

Next, we investigated that the integrity of each individual dataset had been maintained by examining the STARmap and SNARE-seq cells individually by their original labels (Fig. 6d, e). There is clear alignment between the original cell labels of each dataset, indicating that the integration defined the metagenes relevant to specific cell populations for both datasets. This suggests that the unshared features can be included into the integration without unduly dominating the metagene calculations.

**Fig. 7:**
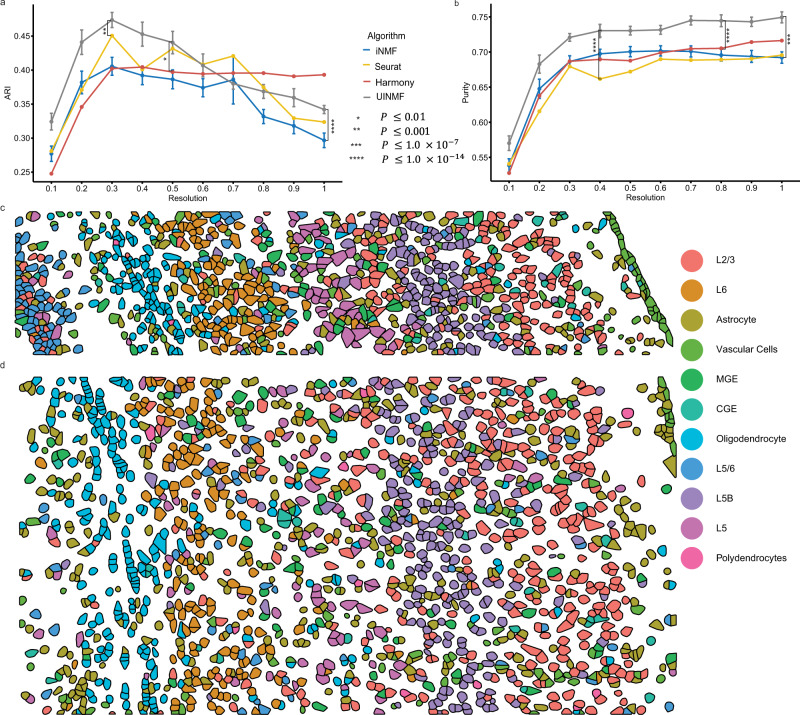
Incorporating unshared chromatin and gene features improves integration of spatial transcriptomic and multimodal data. We compared UINMF results with those from iNMF (*P* = 5.934 × 10^−15^, *P* = 1.046 × 10^−11^)), Harmony (*P* = 0.002927, *P* = 2.2 × 10^−16^), and Seurat v3 (*P* = 0.0004184, *P* = 2.2 × 10^−16^) using ARI (**a**) and purity (**b**) metrics. Comparisons were evaluated using a paired, one-sided Wilcoxon test (*n* = 200 values for each test, with each sample having 10 values across 10 resolutions). For nondeterministic algorithms, data are presented as mean values +/− SEM. We also confirmed that the spatial arrangement of predicted cell types in both STARmap replicate one (**c**) and replicate two (**d**) matches the known organization of the cortex.

To quantify the derived benefit of including the additional features into the analysis, we then calculated the purity and ARI scores for ten initializations (Fig. 7a, b). UINMF significantly outperformed the iNMF algorithm on both ARI (*P =* 5.934 × 10^−15^, paired one-sided Wilcoxon test) and purity (*P* = 1.046 × 10^−11^, paired one-sided Wilcoxon test). The iNMF algorithm has an ARI competitive with that of UINMF at only a single louvain resolution (0.7). At this resolution, UINMF still has a superior purity score, substantiating the benefit of including additional features using the UINMF algorithm.

Next, we benchmarked the UINMF method against Seurat v3 and Harmony. In comparison to Seurat v3, UINMF has a significantly better ARI score across resolutions (*P* = 0.0004184). Additionally, UINMF also has superior purity over Seurat v3 across all tested resolutions (*P* = 2.2 × 10^−16^, paired one-sided Wilcoxon test). Similarly, UINMF has a significantly higher purity score (*P* = 2.2 × 10^−16^) at all Louvain resolution in comparison to Harmony. UINMF also has a significantly higher average ARI score than Harmony at all Louvain resolutions (*P* = 0.002927). While Harmony does achieve higher ARI scores at the higher Louvain resolutions, it is critical to note that these are clearly not optimal values for this dataset. Taken together, these results show that the use of UINMF to incorporate added features to the data integration significantly improves the integration of single-cell multimodal data and spatial transcriptomic data.

Because a key motivation for integrating the multimodal and spatial transcriptomic data was bringing enhanced resolution within the context of spatial coordinates, we next plotted the results of the UINMF integration in space. Applying the cell type labels from UINMF to STARmap replicate one (973 cells, Fig. 7c) and replicate two (1549 cells, Fig. 7d), we found that the UINMF results accord well with the cortical structure. The excitatory neurons are arranged in layers, with L6, L5, and L2/3 clearly visible. Likewise, we also can identify the oligodendrocyte-rich white-matter below the cortex. Additionally, the vascular cell population, which contains endothelial and mural cells, comprise the outermost group of cortical cells.

The SNARE-seq and STARmap integration leverages two distinct types of unshared features, as the *U* matrix contains both unshared genes as well as snATAC-seq peak information. To determine how the unshared features from each modality contribute to the integration, we repeated the integration using only one type of unshared data. Specifically, we integrated the SNARE-seq and STARmap datasets using UINMF, and only the unshared genes (2688 genes) as unshared features. We also integrated the SNARE-seq and STARmap datasets using UINMF, and only the unshared peaks (1431 peaks) as unshared features (Supplementary Fig. 5). The results indicate that the unshared genes contribute most substantially to the improvement in data integration, but that using the peak data individually and additively enhances the final integration result.

### Incorporating nonorthologous genes improves the integration of cross-species data

**Fig. 8:**
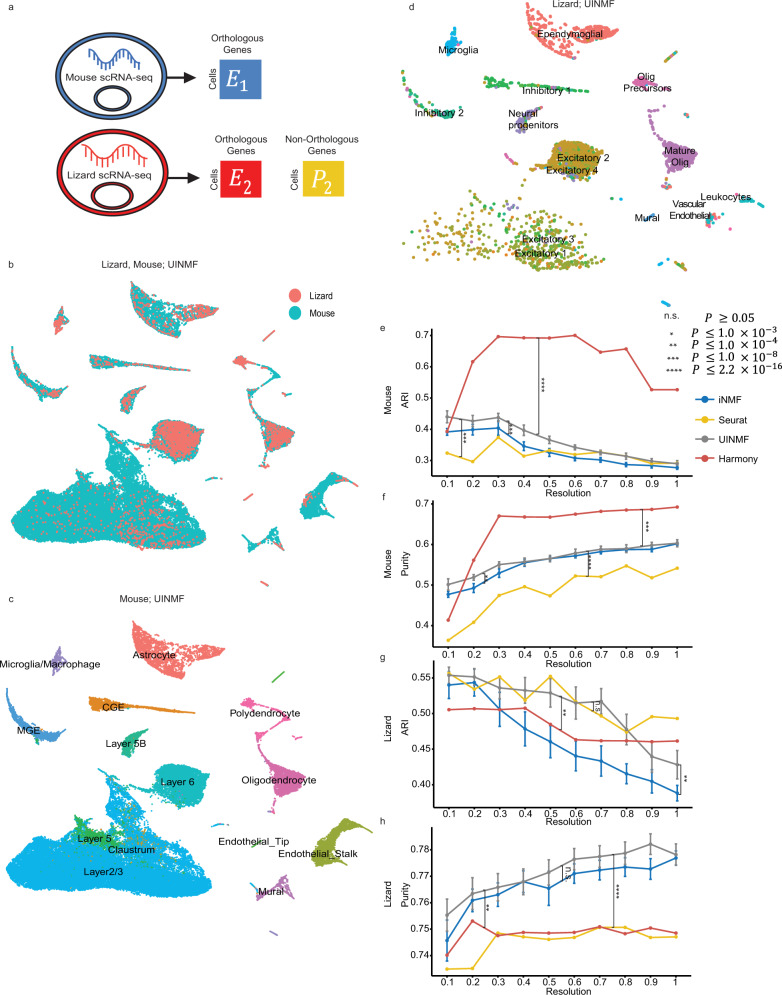
The inclusion of non-orthologous genes improves the integration of cross-species data. We use UINMF to include both orthologous and non-orthologous genes when integrating the datasets (**a**), and demonstrate the alignment between the two datasets (**b**). We also confirmed cell type correspondence by examining only the mouse cells (**c**) and only the lizard cells (**d**), both labeled with their published cell labels. To show the advantage of including the non-orthologous genes, we show the difference in ARI (**e**) and purity (**f**) scores using the originally published mouse labels, comparing UINMF performance to iNMF (*P* = 3.626 × 10^−9^, *P* = 6.258 × 10^−4^), Seurat (*P* < 2.2 × 10^−16^, *P* = 3.047 × 10^−11^), and Harmony (*P* < 2.2 × 10^−16^, *P* = 2.815 × 10^−12^). We compare algorithm performance using a paired, one-sided Wilcoxon test, where *n* = 200 ARI (purity) measures. We also confirm a similar trend in the ARI (**g**) and purity (**h**) scores using the original lizard labels to assess performance differences between UINMF and iNMF (*P* = 1.145 × 10^−6^, *P* = 0.07157), Seurat (*P* < 2.2 × 10^−16^, *P* = 0.8148), and Harmony (*P =* 1.674 × 10^−5^, *P* < 2.2 × 10^−16^). For nondeterministic algorithms, data are presented as mean values +/− SEM.

Previous integrations of cross-species datasets have been limited to genes that are orthologous between species, as non-orthologous genes, by definition, are not shared between datasets^25^. Yet, non-orthologous genes can be key marker genes within a species, providing crucial information for distinguishing cell populations. Using UINMF, we were able explore the potential benefits of including non-orthologous genes in cross-species integration. For this cross-species analysis, we integrated scRNA data (4187 cells) from the pallium of the bearded dragon lizard (*Pogona vitticeps)*^37^ with scRNA data from the mouse frontal cortex (71,639 cells)^29^. We first selected 1979 variable genes that were annotated as one-to-one orthologs between the two species. Then we selected an additional 166 non-orthologous variable genes from the lizard dataset. We integrated the datasets using UINMF, with the one-to-one orthologs as shared features, and the non-orthologous genes from the lizard as unshared features (Fig. 8a). UINMF successfully aligned the two datasets, as illustrated by the overlapping distributions of the two datasets within the UMAP space (Fig. 8b). To confirm the correspondence between the cell types of the two species, we plotted only the mouse cells (Fig. 8c) and only the lizard cells (Fig. 8d), colored by their originally published labels. Strong correspondence between cell types, including excitatory neurons, inhibitory neurons, and non-neuronal cells, can be observed.

To examine the additional benefit of including non-orthologous genes when performing cross-species integrations,we performed the integration using iNMF to establish a baseline. The baseline iNMF integration was limited to the 1979 orthologous genes, and resulted in a lower quality integration (Supplemental Fig. 6). The mural cell populations had decreased alignment between the two species, and many of the astrocytes were misaligned to the excitatory neuron clusters. Furthermore, the lizard’s excitatory neuron subtypes were not distinctly separated.

In order to quantify the advantage of UINMF over iNMF in cross-species integrations, we compared the purity and ARI scores of the two algorithms across ten initializations. To ensure that any differences observed were consistent for both species, we used both the published mouse cell labels, as well as the published lizard labels, to benchmark the algorithms. Including the non-orthologous genes using UINMF resulted in a significant increase in both the ARI (*P =* 3.626 × 10^−9^, paired one-sided Wilcoxon test) and the purity (*P* = 6.258 × 10^−4^, paired one-sided Wilcoxon test) of the mouse dataset (Fig. 8e, f). We also noted a significant increase in the ARI (*P* = 1.145 × 10^−6^, paired one-sided Wilcoxon test) of the lizard data set (Fig. 8g). Although UINMF does not show a significant increase in the purity (*P* = 0.07157, paired with one-sided Wilcoxon test) of the lizard dataset, UINMF is able to achieve a higher maximum purity score at most resolutions (Fig. 8h), and shows significant improvement in all other measures of integration quality.

We also compared the Purity and ARI scores of UINMF to those achieved by Seurat v3 and Harmony. UINMF shows a significant improvement in purity scores over Seurat v3 using both mouse (*P* < 2.2 × 10^−16^, paired one-sided Wilcoxon test) and lizard (*P* < 2.2 × 10^−16^, paired one-sided Wilcoxon test) labels. When comparing ARI scores, there is not a significant difference between Seurat v3 and UINMF using the lizard labels (*P* = 0.8148, paired one-sided Wilcoxon test); however, the mouse labels show a significant improvement (*P* = 3.047 × 10^−11^, paired one-sided Wilcoxon test). Therefore, UINMF significantly outperforms Seurat v3 on three out of the four measures assessed. When examining the difference in performance between UINMF and Harmony, Harmony shows significantly improved ARI (*P* < 2.2 × 10^−16^, paired one-sided Wilcoxon test) and purity (*P* *=* 2.815 × 10^−12^, paired one-sided Wilcoxon test) scores using the mouse labels. However, UINMF has superior performance when comparing the ARI (*P* = 1.674 × 10^−5^, paired one-sided Wilcoxon test) and purity (*P* < 2.2 × 10^−16^, paired one-sided Wilcoxon test) scores using the lizard labels. Harmony’s increased performance using the mouse labels, but decreased performance when using the lizard labels, leads us to believe that Harmony has learned principal components driven primarily by the mouse dataset, neglecting to learn factors that likewise distinguish the lizard cells. The large difference in dataset size between the mouse (71,639 cells) and lizard (4187 cells) supports this hypothesis. UINMF innovatively learns joint metagene factors, resulting in a final integration that respects biological factors in both datasets.

A nice property of UINMF is its flexibility for a range of types of integration analyses, including those with more than two datasets. To demonstrate a case where this is useful, we performed a cross-species integration of the excitatory neurons of the primary motor cortex data of the human (7805 cells), mouse (8242 cells), and the marmoset (8166 cells)^38^.

Using 2757 orthologous genes shared between all three species as shared features, we then selected 353 non-orthologous mouse genes, 290 non-orthologous marmoset genes, and 671 non-orthologous human genes as unshared features. UINMF successfully aligned the three datasets (Supplemental Fig. 7a), while preserving the originally labeled cell types of each species (Supplemental Fig. 7b–d). Interestingly, however, there were two distinct populations of mouse neurons, L5 ET and L5/6 NP, that do not align to the primate data.

We further investigated these clusters to understand why they did not align. The original publication similarly identified primate-specific features of L5 neurons. Specifically, the L5 ET neurons for all species showed cell-type-specific expression of certain ion channel subunits, but the L5 ET neurons of the primates had increased cell-type-specific expression of potassium and calcium channels compared to the mouse L5 neurons. It was hypothesized that these differences drove the observable differences in physiological response between the mouse and primate ET neurons. We thus checked whether our non-aligning clusters reflect these species differences in ion channel expression. We compared the mouse cells that successfully aligned with the primate L5 ET neurons (Cluster 9), with those L5 ET mouse cells that were unaligning (Cluster 13). We observed that the ion channel subunits previously annotated^38^ as showing conserved expression across mouse and primates were again consistent in expression, regardless of their alignment status (Supplemental Fig. 7e). However, for genes previously identified as showing primate-specific enrichment in L5 ET cells^38^, we saw a distinct difference in the expression levels between the unaligning and aligning mouse cells, providing biological support for the algorithmic separation of these clusters (Supplemental Fig. 7f).

## Discussion

We have extended our previous integrative nonnegative matrix factorization algorithm^25^ by adding an unshared feature matrix. This addition accommodates features that are not present in all datasets and increases the amount of information that is used to define the metagenes. We showed that the inclusion of unshared features provides clear advantages across four different types of integration analyses. First, UINMF can be used to incorporate intergenic information when integrating transcriptomic and epigenomic datasets. Second, UINMF can incorporate genes not measured in targeted spatial transcriptomic datasets, allowing better resolution of fine cellular subtypes within a spatial coordinate frame. Third, UINMF can utilize all of the information present in single-cell multimodal datasets when integrating with single-modality datasets. Additionally, UINMF can accommodate non-orthologous genes in cross-species integration analyses.

A recent preprint described MultiMAP, another single-cell data integration approach that can incorporate unshared features. Our approach differs from MultiMap in several key ways. First, MultiMAP uses singular value decomposition followed by nonlinear dimensionality reduction^39^. Therefore, the resulting reduced dimensions are not biologically interpretable. In contrast, UINMF learns factor loadings that represent metagenes, groups of genes that often reflect an underlying biological process or technical artifact. Second, MultiMAP makes a key assumption that the data is uniformly distributed on the latent manifold. While this assumption may hold at times when the datasets to be integrated are sampled from the same tissue, several common integration scenarios violate this assumption. For example, nuclear isolation protocols and whole-cell dissociation protocols each have different cell-type-specific biases, leading to different cell type distributions. Similarly, cross-species integrations may present a challenge if the proportions of homologous cell types are different. In contrast, UINMF does not rely on the uniform distribution assumption.

With the rapid development of multimodal and spatial transcriptomic technologies, we anticipate that the UINMF algorithm will prove useful for a wide variety of analyses. As the additional *U* matrix can incorporate any type of cellular features, with no assumptions about their relationship to gene-centric features, the algorithm is inherently flexible to accommodate a variety of data types and modalities. Future applications could examine the incorporation of data types such as Hi-C^40^ measurements, as well as the potential to use UINMF on a diverse collection of in situ hybridization and immunohistochemistry datasets with limited numbers of genes. We expect that, as additional experimental methods for single-cell measurement are developed, our approach will prove increasingly useful for a broad variety of single-cell integration tasks.

## Methods

We extend our previously published ANLS algorithm for solving the iNMF problem^25^ so that we can now incorporate unshared features when integrating across datasets. The unshared feature matrix can accommodate any type of unshared feature, whether gene-centric or otherwise. In this paper, we incorporated intergenic peaks from snATAC-seq data and additional genes not measured in all datasets, although many other applications are possible.

For each data set *E*^1^, *E*^2^, ....*E*^*n*^, we normalize the data, and select *m* variable genes (shared across all datasets), and *z*_*i*_ variable features (not shared across all datasets), such that after scaling ${E}^{i}{\,}\epsilon{\,} {{{\mathbb{R}}}_{+}}^{{n}_{i}\times (m+{z}_{i})}$ (*i* = 1,....,*N*). For a given *K*, the user-defined number of metagenes, and *λ*_*i*_, the UINMF optimization problem is:

1 $${{{{{{\rm{argmin}}}}}}}_{{H}^{i}\ge 0,W\ge 0,{V}^{i}\ge 0,{U}^{i}\ge 0}\mathop{\sum }\limits_{i}^{d}{\left\Vert\left({E}^{i}{P}^{i}\right)-{H}^{i}\left(\left(W0\right)+\left({V}^{i}{U}^{i}\right)\right)\right\Vert}_{F}^{2}+{\lambda }_{i}\mathop{\sum }\limits_{i}^{d}{\Vert{H}^{i}\left({V}^{i}{U}^{i}\right)\Vert}_{F}^{2}$$

where *P*^*i*^ is the unshared feature matrix, *H*^*i*^ are the cell factor loadings, *W* is the shared metagenes, *V*^*i*^ are the dataset-specific, shared metagenes, and *U*^*i*^ are the dataset-specific, unshared metagenes.

We then derive a coordinate block descent (BCD) algorithm^41^ for solving the UINMF optimization problem. The BCD approach divides the parameters into blocks, and then finds the optimal parameters by updating each block while holding the others fixed. Because each block-wise optimization sub-problem is convex, iterating these updates is guaranteed to converge to a local minimum^41^. To solve the UINMF optimization problem, we use matrix blocks, one block for each of ${H}^{i}\epsilon {{{\mathbb{R}}}_{+}}^{{n}_{i}\times k}$, $W\epsilon {{{\mathbb{R}}}_{+}}^{k\times m}$, ${V}^{i}\epsilon {{{\mathbb{R}}}_{+}}^{k\times m}$, and ${U}^{i}\epsilon {{{\mathbb{R}}}_{+}}^{k\times {z}_{i}}(i=1,....,N).$

Each sub-problem is a nonnegative least squares optimization, which we solve numerically using an efficient C++ implementation of the block principal pivoting algorithm^42^. To update *H*^*i*^, we solve

2 $${H}^{i}={{{{{{\rm{arg }}}}}}{{{{{\rm{min }}}}}}}_{H\ge 0}\left\Vert\left(\begin{array}{c}\left(\begin{array}{c}{{W}^{T}}\\ {{0}^{{z}^{i}\times k}}\end{array}\right)+\left(\begin{array}{c}{{V}^{{i}^{T}}}\\ {{U}^{{i}^{T}}}\end{array}\right)\\ \sqrt{{\lambda }_{i}}\left(\begin{array}{c}{{V}^{{i}^{T}}}\\ {{U}^{{i}^{T}}}\end{array}\right)\end{array}\right){{H}^{i}}^{T}-\left(\begin{array}{c}{{E}^{i}}\\ {{0}^{{n}_{i}\times \left(m+{z}^{i}\right)}}\end{array}\right)^{T}\right\Vert_{F}^{2}$$

while holding the other parameters fixed. Similarly, to update the other parameters, we solve the following subproblems:

3 $$W={{{{{{\rm{arg }}}}}}{{{{{\rm{min }}}}}}}_{W\ge 0}{\left\Vert\left(\begin{array}{c}{{H}^{1}}\\ {{H}^{i}}\end{array}\right)W-\left(\begin{array}{c}{{E}^{1}-{H}^{1}{V}^{1}}\\ {{E}^{i}-{H}^{i}{V}^{i}}\end{array}\right)\right\Vert}_{F}^{2}$$

4 $${V}^{i}={{{{{{\rm{arg }}}}}}{\min }}_{V\ge 0}{\left\Vert\left(\begin{array}{c}{{H}^{1}}\\ {\sqrt{{\lambda }_{i}}{H}^{d}}\end{array}\right){V}^{i}-\left(\begin{array}{c}{{E}^{1}-{H}^{1}W}\\ {{E}^{i}-{H}^{i}W}\end{array}\right)\right\Vert}_{F}^{2}$$

5 $${U}^{i}={{{{{{\rm{arg }}}}}}{{{{{\rm{min }}}}}}}_{U\ge 0}{\left\Vert\left(\begin{array}{c}{{H}^{1}}\\ {\sqrt{{\lambda }_{i}}{H}^{d}}\end{array}\right){U}^{i}-\left(\begin{array}{c}{{P}^{i}}\\ {{0}^{{n}_{i}\times {z}^{i}}}\end{array}\right)\right\Vert}_{F}^{2}$$

We iterate these updates until convergence, which we determine by calculating the decrease in the objective function at the conclusion of each iteration (Supplemental Fig. 8). We consider the algorithm to have converged when the decrease in the objective value function between the previous and current iteration, weighted by dividing by their mean, is less than the epsilon parameter set by the user. For all UINMF analyses in this paper, we set the convergence threshold to *ε* = 1.0 × 10^−10^ and set the maximum number of iterations to 30 for each analysis, unless otherwise noted.

Note that iNMF uses a constant penalty term *λ* for all datasets. When implementing UINMF, however, we introduced a separate *λ*_*i*_ parameter for each dataset, such that the penalty applied to *E* is weighted by *λ*_1_, *E*_2_ has a regularization weight of *λ*_2_, etc. The inclusion of *λ*_*i*_ allows for the tuning of dataset penalization at the user’s discretion. We found that in some cases, using a different penalty value for different datasets could improve results. However, we simply used *λ*_*i*_ = 5 (the default value in our iNMF and UINMF implementations) for all analyses except the STARmap and DROPviz integration. The STARmap and DROPviz integration does achieve the best results with different *λ* values for each dataset.

### Increase in Computational Complexity

The difference in computational complexity between UINMF and iNMF increases with the number of unshared features used in UINMF, but the difference between the runtime of the two algorithms is not prohibitive in practice (Supplemental Figs. 9, 10). To assess the theoretical difference in computational complexity between the algorithms, assume the same total number of features are present in the datasets input into each algorithm. Let iNMF operate on a dataset that has *g* shared features, and let *g* = *m* + *z*, where *m* is the number of shared features and *z* is the number of unshared features of the UINMF dataset. Let *K* be the user-defined number of metagenes. For each iteration, UINMF solves for U^*z* × *K*^ and V_*i*_^*m* × *K*^ separately, but iNMF performs the same number of calculations to solve for ${{V}_{i}}^{g\times K}$, since *g* = *m* + *z*. When solving for the shared metagene matrix, *W*, iNMF solves the optimization problem for a *g* × *K* matrix, whereas UINMF must only solve a *m* × *K* matrix. Because the shared metagene matrix has less features in UINMF (*g* > *m*), each iteration of the algorithm actually constitutes less computational complexity than iNMF given the same total number of features.

### Evaluating Time and Memory Usage

To benchmark the memory usage and run time of UINMF in comparison to current methods, we measured the performance of Seurat v3, Harmony, iNMF, and UINMF when integrating datasets of 20,000, 40,000, 60,000, 80,000, and 100,000 cells. To obtain datasets with these cell populations, we sampled equivalent numbers of cells from the Dropviz scRNA-seq and STARmap datasets. As there are only 32,845 cells in the STARmap dataset, for the 80,000 and 100,000 cells data integrations, we sampled the remaining cells all from the DROPviz dataset. The integrations for the four algorithms were performed on the same sample datasets. We used the 28 shared genes between the datasets as the shared features for all four algorithms. We set *λ* = 5 and *K* = 27 as the parameter for both algorithms. Using UINMF, we incorporated an additional 2775 unshared genes when performing the dataset integration. We ran each iNMF and UINMF analysis 5 times to assess run-to-run variation in time and memory usage but found that these numbers were very stable. For the Seurat v3 integration, the number of dimensions was set to 27 for both the *FindIntegrationAnchors* (Seurat 4.0.0) as well as the *IntegrateData* (Seurat 4.0.0) functions. The number of principal components within *RunPCA* (Seurat 4.0.0) was also set to 27 for Harmony as well as Seurat v3 (Supplemental Fig. 1).

### Evaluation Metrics

The alignment score, based on Butler et al. (9), is a measure that captures how well two samples align uniformly within a latent space. A score closer to zero indicates a poor alignment, or mixing of the two samples, whereas a score closer to one is indicative of datasets that share underlying cell types.

Fraction of Samples Closer Than the True Match (FOSCTTM) scores measures how closely two measurements of the same cell are placed within the latent space^3^. We calculate the FOSCTTM score by finding the distance between the scRNA-seq cell and the snATAC-seq label for each barcode. We then divide by the total number of barcoded cells to derive the average FOSCTTM score.

Cluster purity is calculated based on a reference clustering. Each cluster is assigned a type based on the predominant label for that cluster. The cells that correspond correctly to this label are counted. We calculate purity by summing the correct number of labels across all clusters, and dividing by the total number of labeled cells present. Consequently, a score closer to 0 indicates that the cells are not being accurately grouped into clusters by cell types, and a score of 1 indicates perfect grouping by cell type.

To calculate the Adjusted Rand Index (ARI), we measured the similarity between two clusterings by whether we observe matching or non-matching clustering between pairs of samples. The ARI score can range from 0 (no matches) to 1 (perfect matching).

### Integration of RNA and ATAC profiles from SNARE-seq

For a baseline, we integrated the scRNA and snATAC datasets that resulted from the original SNARE-seq publication^2^ with iNMF, using the top 2589 variable genes and their associated snATAC peaks. We took the best optimization of 5 random initializations, each performed with *K* = 30 and *λ* = 5, across ten random seeds. We performed quantile normalization with parameter *knn_k* = 20. We then calculated the average FOSCTTM and alignment scores for each of the 10 random seeds. To assess the additive properties of including the intergenic peaks into the integration, we used the *U*-matrix to hold 1000, 2000, 3000, 4000, 5000, 6000, and 7000 of the top variable intergenic bins. To select these bins, we binned the genome into sections of 100,000 bp. We counted the number of peaks within each bin, where a peak was considered to be inside a bin if over 50% of the peak’s base pairs overlapped the bin. We then filtered the bins, removing bins that were empty, or overlapping with ENCODE Blacklisted regions^43^, genes or promoters. After normalization, we then used the *FindVariableFeatures* function (Seurat 4.0.0) to select the top variable bins. We saw the greatest improvement in performance using 7000 intergenic bins, as this allowed the capture of a large amount of the data variance, without oversaturating the factor loadings with noise. We performed UINMF with *K* = 30, *λ* = 5, and *knn_k* = 20. Using a paired, one-sided Wilcoxon test, we compared the FOSCTTM and alignment scores of UINMF and iNMF.

To compare UINMF performance to that of Seurat v3 and Harmony, we integrated the two datasets using the 2589 shared features. For Seurat v3, we used a PCA dimension of 30. To compare UINMF and Seurat v3 performance, we performed a paired, one-sided Wilcoxon test on the alignment and FOSCTTM scores of UINMF and Seurat v3.

Similarly, to run Harmony, we used the 2589 shared features and 30 principal components for *RunPCA* (Seurat 4.0.0). We also used 30 neighbors for the *FindNeighbors* function. We calculated the alignment and FOSCTTM, and then performed a paired, one-sided Wilcoxon test to assess the difference between UINMF and Harmony performance. To cluster just the RNA and ATAC cells, we similarly used *K* = 30, *λ* = 5, *knn_k* = 100, and a Louvain resolution of 1. To derive ground truth cell type labels, we clustered and annotated the RNA-seq cell barcodes using marker genes. We use these consistent labels as ground truth labels for each UMAP presented in Fig. 2.

### Integration of scRNA-seq and STARmap

We integrated the STARmap spatial transcriptomic dataset (31,294 cells, filtered for hippocampus and claustrum cells) and a scRNA-seq dataset (70,514 cells (1125 removed for non-expressing)) using iNMF. For this iNMF analysis, we were limited to the 28 genes measured in the STARmap dataset. Both iNMF and CCA/PCA (used by Seurat v3) are limited to no more components than the number of genes, while UINMF can estimate more dimensions because it also incorporates unshared genes. We thus used *K* = 27 dimensions for both iNMF and Seurat v3. For iNMF we also used *λ* = 5, and a quantile normalization with K-nearest neighbors of 20. Using UINMF, we included an additional 2775 of the most variable genes. The parameters for the UINMF integration were *K* = 40 and *λ* = 10 for the STARmap data and *λ* = 1 for the scRNA-seq data; *knn_k* = 20 for the quantile normalization; and Louvain resolution of 1. For both iNMF and UINMF, we perform 5 initializations with the same random seed and pick the best one. We calculate the cluster purity for both algorithms using the scRNA-seq labels, and use the highest number of cells present to annotate the clusters by cell type. These annotations were then applied within the context of 3D space using the originally provided STARmap coordinates. For five different random seeds, we measured the difference between the cluster purity and ARI of iNMF and UINMF using a paired, one-sided Wilcoxon test.

For Seurat v3, we similarly used the 28 shared genes and a PCA dimension of 27. We then calculated the Purity and ARI scores at each Louvain resolution from 0.1 to 1, in increments of 0.1. We performed a paired, one-sided Wilcoxon test to assess the difference between UINMF and Seurat v3 performance quantified by purity and ARI scores.

Similarly, to run Harmony, we used the 28 shared genes and 27 principal components for *RunPCA* (Seurat 4.0.0). We also used 27 as the number of dimensions used for the *FindNeighbors* function. For each Louvain resolution from 0.1 to 1, in increments of 0.1, we calculated the purity and ARI scores. We then performed a paired, one-sided Wilcoxon test to assess the difference between UINMF and Harmony performance quantified by purity and ARI scores.

To analyze the effect of the additional features on the memory usage and run time of the algorithm, we performed five initializations for 0, 1000, 2000, 3000, and 4000 extra features (Supplemental Fig. 9). For consistency, we used *λ* = 5 and *K* = 27 for all time and memory analyses.

After performing the dataset integration, it was possible to use *imputeKNN*, with *knn*_*k* = 20, to impute gene values for genes originally unmeasured by the STARmap technology. By using the gene profiles of the closest scRNA cells to impute probable gene expression values for the STARmap cells, we are able to predict expression for genes not originally assayed with the 3D tissue space. Specifically, we show the results of KNN imputation for the *Trf* and *Dcn* genes (Supplemental Fig. 4.)

To examine the individual contribution of each factor (*V*^*i*^, *U*, *W*) to the matrix reconstruction, we develop the *calcNormLoadings* function. Since $W\epsilon {{{\mathbb{R}}}_{+}}^{k\times m}$, ${V}^{i}\epsilon {{{\mathbb{R}}}_{+}}^{k\times m}$, and ${U}^{i}\epsilon {{{\mathbb{R}}}_{+}}^{k\times {z}_{i}}$are characterized by different numbers of features, we standardize the factor loadings by dividing by the number of features pertinent to each matrix. For each factor *l* in *K*, we measure the contribution to reconstruction as

6 $${U}_{l}={{||H}[,l]\times {{{{{{\rm{U}}}}}}}^{i}[l,]{||}}_{F}^{2}/{z}_{i}$$

7 $${W}_{l}={{||H}[,l]\times {W}^{l}[l,]{||}}_{F}^{2}/m$$

8 $${{V}^{1}}_{l}={{||H}[,l]\times {V}^{1}[l,]{||}}_{F}^{2}/m$$

9 $${{V}^{2}}_{l}={{||H}[,l]\times {V}^{2}[l,]{||}}_{F}^{2}/m$$

The resulting ranked norms provide intuition as to which factors each matrix contributes most strongly to (Supplemental Fig. 3).

### Integration of scRNA-seq and osmFISH

Using the osmFISH dataset (33 genes, 6471 cells) we excluded hippocampal and cells from the top left of the tissue slice originally labeled “excluded” in the original osmFISH publication, as well as cells with zero detected genes. A total of 5185 cells passed these filtering criteria. The scRNA-seq dataset, originally 71,639 cells, was reduced to 70,020 (1,619 cells non-expressing for the 33 genes of interest were removed). We ran the  iNMF algorithm using the 33 shared genes, *λ* = 5, *K* = 32, and took the best optimization of 5 random initializations for each of the 10 seeds. Note that, as with the STARmap data, iNMF and Seurat v3 are limited to no more components than the number of genes, while UNIMF can estimate more factors due to the use of unshared genes. Using UINMF, we integrated the osmFISH spatial transcriptomic data with the scRNA-seq using the 33 shared genes as well as the 2000 most variable unshared genes. We used 10 different random seeds, with *λ* = 5 and *K* = 40, and took the best optimization of 5 random initializations for each seed. We calculated the Purity and ARI for each algorithm at Louvain resolutions 0.1 through 1.0, in 0.1 increments. We used the published DROPviz labels as our reference clustering, and assessed the significance of our findings using a paired, one-sided Wilcoxon test. We used *knn_k* = 150 for the *quantile_norm* function (rliger 1.0.0), *n_neighbors* = 150 for the *runUMAP* function (rliger 1.0.0), and a resolution of 0.5 for Louvain community detection (rliger 1.0.0).

For the Seurat v3 integration, we also used the 33 shared genes and a PCA dimension of 32. Using louvain resolutions from 0.1 to 1.0 in 0.1 increments, we assessed the performance of Seurat v3 using purity and ARI scores. We compared Seurat v3 and UINMF using a paired, one-sided Wilcoxon test.

Using Harmony, we integrated the two datasets using the 33 shared genes and 32 as the number of principal components and dimensions for the *RunPCA* and *FindNeighbors* functions (Seurat 4.0.0). We quantified Harmony performance by calculating purity and ARI scores for louvain resolutions from 0.1 to 1.0 in 0.1 increments, and compared the results against UINMF using a paired, one-sided Wilcoxon test.

### Cross-Species Integration

The Lizard Pallium dataset^37^ originally had 4202 cells, but we limited the cells used to the 4187 cells deemed high quality in the original publication. We integrated this dataset with the scRNA-seq dataset from the mouse brain^29^ (71,639 cells). We used the original publication’s 1-to-1 ortholog labels to select orthologs common to both the mouse and lizard dataset. Using these as our shared features, we normalized and scaled the data. We then selected 1979 shared genes, using a variance threshold of 0.3. For UINMF, we used the same variance threshold to select 166 non-orthologous genes from the lizard. We optimized UINMF and iNMF with *K* = 30, *λ* = 5, and took the best of 5 random initializations for 10 random seeds. We performed quantile normalization for each optimized object using the mouse dataset as a reference. To ensure that any differences in purity and ARI score were not driven by a single species, we calculated the ARI and Purity scores using both the lizard and the mouse cell labels separately as ground truth. We performed these calculations for each of the ten seeds at louvain resolutions from 0.1 to 1.0 in increments of 0.1. To examine the difference between UINMF and iNMF performance, we performed a paired, one-sided Wilcoxon test between the ARI and Purity values, for the mouse and lizard labels, respectively. When generating the UMAPs shown, we used the default louvain resolution of 0.25, and the default nearest neighbors of 10.

For Seurat v3 integration, we used the 1979 highly variable, orthologous genes identified between the datasets, and a PCA dimension of 30. Using louvain resolutions from 0.1 to 1.0 in 0.1 increments, we assessed the performance of Seurat v3 using purity and ARI scores with both the lizard and mouse annotations. To compare the performance of Seurat v3 and UINMF, we used a paired, one-sided Wilcoxon test.

Similarly, when evaluating the performance of Harmony in integrating the two datasets, we use the same 1979 orthogolous genes. We use 30 as the number of principal components and dimensions for the *RunPCA* and *FindNeighbors* functions (Seurat 4.0.0). We quantified Harmony performance by calculating purity and ARI scores for louvain resolutions from 0.1 to 1.0 in 0.1 increments, and compared the results against UINMF using a paired, one-sided Wilcoxon test.

### Integration of SNARE-seq and STARmap

To integrate the multi-omic SNARE-seq dataset (10,309 cells) with the spatial transcriptomic STARmap dataset (2522 cells), we used the number of shared genes (944 genes), as well as 4,119 unshared features. To generate the unshared features, we selected genes with a variance threshold higher than that of 0.1, and then removed genes shared between datasets, for a total number of 2688 unshared genes. We selected the chromatin accessibility peaks with a variance greater than 0.01 (1431 peaks). For iNMF benchmarking, we used only the 944 shared genes. We optimized UINMF and iNMF with *K* = 30, *λ* = 5, and took the best of 5 random initializations for 10 random seeds. We performed quantile normalization for each optimized object using the SNARE-seq dataset as a reference. Using the SNARE-seq cell labels as ground truth, we calculated the ARI and Purity scores for each of the ten seeds at louvain resolutions from 0.1 to 1.0 in increments of 0.1. We set nearest neighbors to 100 when generating the UMAPs shown, and the louvain resolution to 1. To assess the difference between UINMF and iNMF functioning, we performed a paired, one-sided Wilcoxon test between the ARI and purity values. The UMAPs shown have a louvain resolution of 1.0, and are labeled by jointly examining the original STARmap and SNARE-seq cell labels.

To quantify the contribution of each type of unshared feature to the resulting improvement in dataset integration quality, we also performed the UINMF integration using only the 1431 snATAC-seq peaks, and using only the 2688 unshared genes. For each analysis, we optimized the UINMF optimization equation using *K* = 30, *λ* = 5, and took the best of 5 random initializations for 10 random seeds. We performed quantile normalization for each optimized object using the SNARE-seq dataset as a reference. Using the SNARE-seq cell labels as ground truth, we calculated the ARI and purity scores for each of the ten seeds at louvain resolutions from 0.1 to 1.0 in increments of 0.1 (Supplemental Fig. 5).

To assess the performance of Seurat v3, we used the 944 shared genes, and set the PCA dimensions equal to 30. We then calculated the purity and ARI scores for each Louvain resolution from 0.1 to 1.0, in increments of 0.1. We conducted a paired, one-sided Wilcoxon test between UINMF and Seurat v3 performance in terms of ARI and purity scores.

Using Harmony to perform the integration, we used the 944 shared genes and the default 30 principal components for *RunPCA* (Seurat 4.0.0). Similarly, we used the default (30) for the number of dimensions in the *FindNeighbors* function (Seurat 4.0.0). Using louvain resolutions from 0.1 to 1.0 in 0.1 increments, we assessed the performance of Harmony using purity and ARI scores. We compared Harmony and UINMF using a paired, one-sided Wilcoxon test on both the purity and ARI scores.

We assessed the impact of the additional features on runtime and memory usage by performing UINMF using 0, 1000, 2000, 3000, and 4000 extra features (Supplemental Fig. 10). Note that performing UINMF with 0 unshared features is equivalent to performing iNMF. For consistency, we used *λ* = 5 and *K* = 30 for all time and memory analyses.

### Assessing the contribution of unshared features

In order to better quantify how the number of shared and unshared features used within an analysis interact to produce the observable differences in the quality of integration, we perform a stepwise analysis. We select two single-cell transcriptomic datasets taken from the mouse Primary Motor Area (MOp)^13^, with two different protocols: SMART-seq and TenX. We then randomly downsampled the TenX dataset such that the datasets had an equivalent number of cells (6288 cells). We then defined the 2500 most variable genes between datasets.

From these 2500 most variable genes, we randomly sampled a set of 500 genes. Using these as shared features, we integrated the datasets using iNMF, letting *K* = 30, and *λ* = 5, selecting the best optimization score from three random initializations. After performing matrix decomposition, we performed quantile normalization using the SMART-seq dataset as our reference, and performed Louvain community detection with a resolution of 0.1. Using the SMART-seq labels as ground truth, we calculated the resulting purity and ARI scores. We then decreased the number of shared genes by 100, and repeated the integration, and metric calculations. We continued this process for 500, 400, 300, 200, and 100 shared genes. We repeated this process for ten different sets of randomly selected held-out genes.

To assess how including the unshared features benefits the dataset integration, we integrate the datasets using UINMF. The same 500 shared genes are used as shared features, and the remaining 2000 variable genes not selected are used as unshared features for the SMART-seq dataset. For consistency, we use *K* = 30, *λ* = 5 as the parameters for UINMF optimization, selecting the optimal optimization of three initializations. Similarly, we perform quantile-normalization with SMART-seq as the reference dataset, and calculate the Louvain community detection with a resolution of 0.1. After calculating the purity and ARI scores, we repeat the UINMF analysis for the same 400, 300, 200, and 100 shared genes, adding the unshared genes to the unshared features, such that the number of total features for the SMART-seq dataset always totals to the 2500 top variable genes. We repeat this process for the ten sets of randomly selected held-out genes used for the iNMF analysis.

Lastly, we repeat the UINMF analysis, except instead of including the unshared features from the SMART-seq dataset, we include the unshared features as part of the TenX dataset. We also repeat this process for the ten sets of randomly selected held-out genes used for the iNMF analysis (Supplemental Fig. 2a, b). Using *n*_*neighbors* = 20, we plotted the resulting UMAPS from each analysis using 500 shared features and only 100 shared features (Supplemental Fig. 2c–f).

### Selecting *K* and *λ*

The default parameter settings for iNMF are *K* = 30 and *λ* = 5. To allow for a fair comparison between iNMF and UINMF, we used these parameters settings for both algorithms when performing the SNARE-seq integration with intergenic peaks, the SNARE-seq integration with the 1020 gene STARmap dataset, and the cross-species analysis. For the spatial transcriptomic dataset integrations using osmFISH (33 genes) and STARmap (28 genes), iNMF required the selected *K* to be less than the number of genes. Since one of the advantages of our algorithm is that it does not have this constraint, we selected a value of *K* = 40 for both spatial transcriptomics integrations, the largest *K* we could select without severely impacting the alignment scores (Supplemental Fig. 11). To select *λ* for the spatial transcriptomics datasets, we selected *λ* = (10,1) for the STARmap integration with the scRNA-seq data. The higher penalty is assigned to the STARmap data, and the use of the vectorized lambda showed improved alignment scores over five initializations (Supplemental Fig. 12). We also showed the results of using *λ* = 5 for the osmFISH dataset in order to highlight that default choice of lambda still provides a significant improvement compared to iNMF, and that the results were not driven by the use of a vectorized *λ* (Supplemental Fig. 12).

### Integration of three species

To illustrate the extensibility of the UINMF algorithm to accommodate multiple datasets of unshared features, we performed a cross-species integration of the primary motor cortex data of the human, mouse, and marmoset, sequenced with droplet-based Chromium v3. To elucidate reproducibility and consistency, we downsampled the available excitatory neuron population from each species following the methods of the original publication^38^. This sampling technique resulted in the selection of 8242 mouse excitatory neurons, 8166 marmoset excitatory neurons, and 7805 human excitatory neurons.

To identify non-orthologous and orthologous genes, we downloaded the NCBI table of orthologous genes between species, and identified genes listed as orthologous between all three species, resulting in 14,448 orthologous genes between datasets. Using a variable gene threshold of 0.2, we selected 2757 shared, orthologous genes between the species. To select unshared orthologous genes, we used a threshold of 0.3 for the human data, 0.25 for the mouse, and 0.2 for the marmoset dataset. We used a variable threshold for the non-orthologous genes such that we could retain a relevant population of unshared features for each dataset, despite the differences in sequencing quality and depth. These thresholds resulted in the selection of 353 non-orthologous mouse genes, 290 non-orthologous marmoset genes, and 671 non-orthologous human genes.

We then performed the integration with 3 distinct initializations, keeping the best optimization value, using *K* = 20, *λ* = 10. For quantile normalization, we used the human dataset as a reference, as this was previously reported to have the highest median neuronal gene detection^38^, and used *knn_k* = 50. For Louvain community detection, we used a resolution of 0.25.

### Statistics

All statistical analyses were performed using R (4.0.0). *P* values were calculated using one-sided paired Student *T*-tests or one-sided paired Wilcoxon Rank Sum Tests, as indicated. P-values are reported throughout, with statistical significance considered *P* < 0.05. All error bars represent standard error, with the exception of the box plots in Fig. 2g, h, and Supplementary fig. 2a, b, where the bars represent the highest (lowest) point within 1.5 of the interquartile range.

### Reporting summary

Further information on research design is available in the Nature Research Reporting Summary linked to this article.

## Supplementary information

Supplementary Information

Reporting Summary

## Data Availability

All datasets used in this paper are previously published and freely available. Mouse Frontal Cortex SNARE-seq cells from Chen et al.^2^ are in the GEO database under accession code “GSE126074”. Mouse frontal cortex spatial transcriptomic cells from “Moffit et al. [https://www.starmapresources.org/data]”^10^. Mouse cells from the “somatosensory cortex [http://linnarssonlab.org/osmFISH/]”^12^. Adult mouse brain cells from Saunders et al.^29^ [http://dropviz.org/]. The data is also in the GEO database under accession code “GSE116470”. Lizard Pallium cells from “Tosches, et. al. [https://public.brain.mpg.de/Laurent/ReptilePallium2018/]”^37^. Mouse Primary Motor Cortex (10X and SMARTseq datasets) from “Yao et al. [https://assets.nemoarchive.org/dat-ch1nqb7]”^13^. Mouse, Marmoset, and Human Primary Motor Cortex from “Bakken et al. [http://data.nemoarchive.org/publication_release/Lein_2020_M1_study_analysis/Transcriptomics/sncell/10X/]”^38^. All other relevant data supporting the key findings of this study are available within the article and its Supplementary Information files or from the corresponding author upon reasonable request. Source data are provided with this paper.

## Source data

Source Data

## Code availability

An R implementation of the UINMF algorithm is available as part of the “LIGER package [https://github.com/welch-lab/liger]”. The package is also available on the Comprehensive R Archive Network (CRAN). The package version used for the analyses in the paper has been assigned a citable DOI through Zenodo: 10.5281/zenodo.5762770.
