# Supplementary material for: UINMF performs mosaic integration of single-cell multi-omic datasets using nonnegative matrix factorization
Source: Nat Commun. 2022 Feb 9;13:780. doi: 10.1038/s41467-022-28431-4 (PMC8828882; doi:10.1038/s41467-022-28431-4)
Supplement: Supplementary file 1 — Supplementary Information [file 41467_2022_28431_MOESM1_ESM.pdf]

Supplementary Information

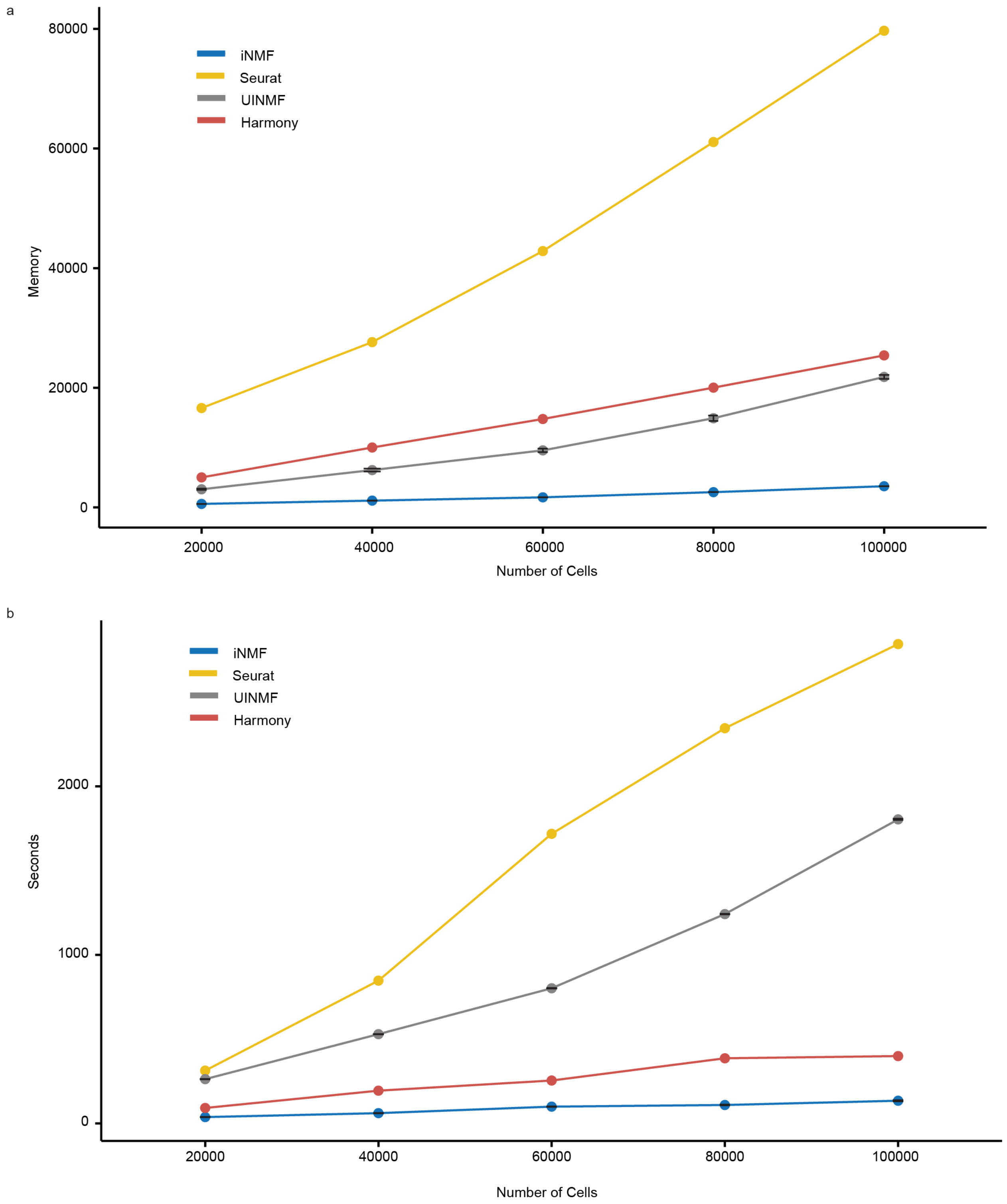

Supplementary Figure 1: Incorporating unshared features does not require prohibitive increases in time or memory usage. Using the STARmap and Dropviz datasets, which have 28

shared genes, we sampled equal numbers of cells from each dataset, taking additional cells from Dropviz once all 32,845 cells from STARmap were sampled. We ran UINMF with an additional 2,775 unshared genes, as shown in Fig. 4, while the other approaches used only 28 features. We benchmarked the increase in peak memory usage (a) and computational time (b) for UINMF, as well as other dataset integration methods that are incapable of including additional features, across an increasing number of cells. Data are presented as mean values +/- SEM.

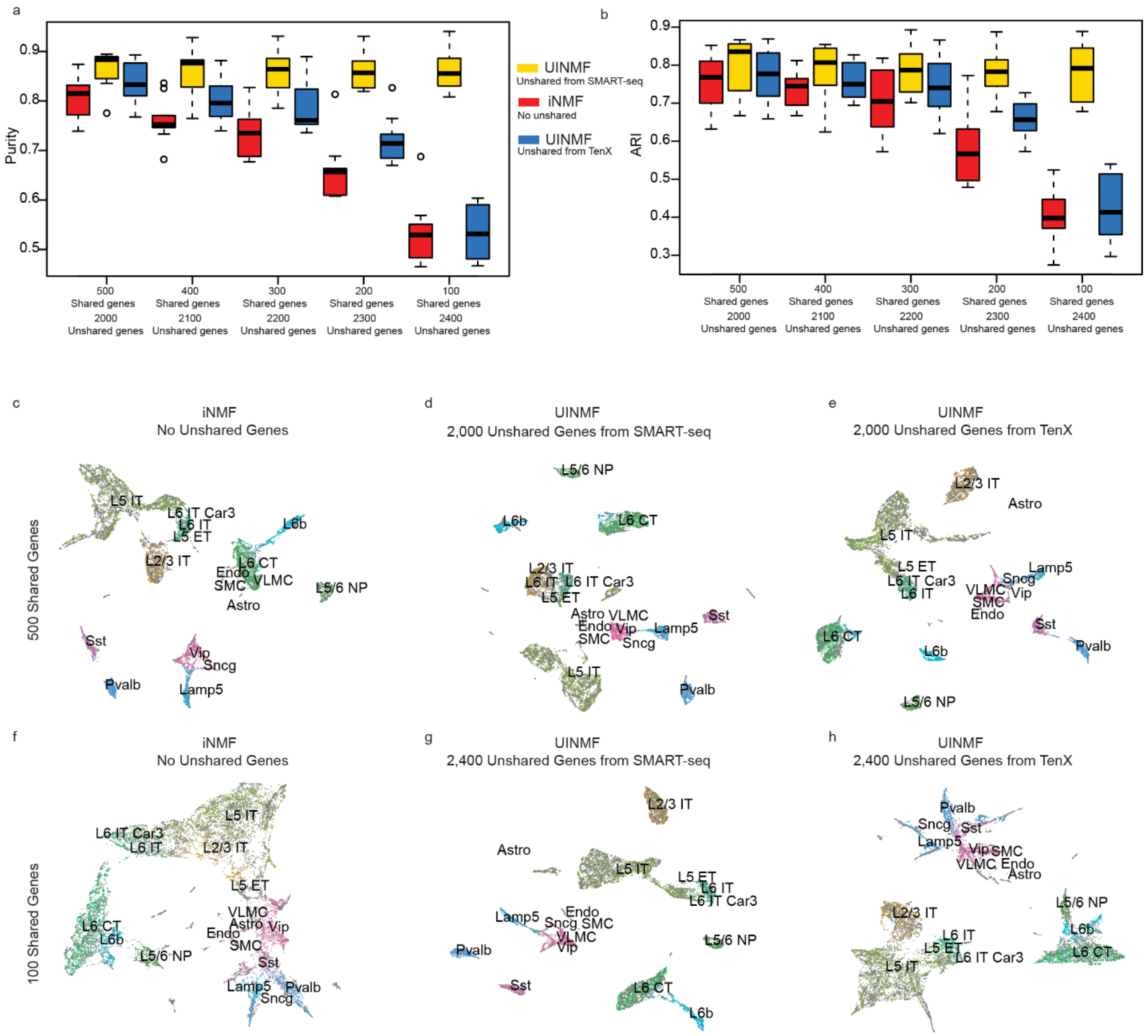

Supplementary Fig. 2. Incorporating unshared genes significantly improves integration performance, particularly as number of shared genes decreases. In terms of both Purity (a) and ARI (b), including the unshared features from the SMART-seq and TenX data preserves higher dataset integration quality as the number of shared features declines. The bold line indicates the median data value, and the boundaries of each box are defined by the first and third data quartiles (25% and 75%, respectively). The upper (lower) whiskers extend from to highest (lowest) point within 1.5 of the interquartile range. Outliers beyond the whiskers are plotted as

points.Including the unshared features from the SMART-seq dataset has the most added benefit, perhaps a result driven by the higher quality of the SMART-seq data. The difference in dataset integration is also visually apparent, as there are clear, distinguishable clusters for all three algorithms using 500 shared features (c,d,e), but with only 100 shared features, iNMF produces a lower resolution UMAP (f). The UINMF algorithm used to include the high quality SMART-seq unshared features maintains substantial integrity, with distinct populations of Lamp5, Pvalb, and Sst cells (g). Lastly, UINMF used to include unshared features from TenX generally maintains higher quality integrations, but is subject to a substantial decline in purity and ARI using only 100 shared features (h).

a

Factor	W Loadings	V^1 Loadings	V^2 Loadings	U Loadings
**1**	9.832	0.001	0.115	0.490
**2**	6.867	0.000	0.685	0.075
**3**	10.572	0.001	0.085	0.071
**4**	5.808	8.698	0.000	0.296
**5**	10.993	0.050	0.679	0.130
**6**	10.218	0.000	0.000	0.347
**7**	8.535	0.000	0.402	0.139
**8**	3.076	44.167	0.000	0.138
**9**	3.236	43.604	0.001	0.198
**10**	8.558	0.000	0.051	0.133
**11**	1.408	0.001	1.092	0.245
**12**	14.251	0.000	0.556	0.061
**13**	8.851	0.000	0.022	0.461
**14**	3.911	0.000	0.436	0.493
**15**	1.579	0.000	1.130	0.360
**16**	6.416	249.953	0.014	0.088
**17**	4.249	0.005	0.179	0.158
**18**	3.575	0.000	0.026	0.288
**19**	10.437	0.002	0.062	0.036
**20**	2.435	0.001	0.546	0.094
**21**	0.622	399.544	0.000	0.254
**22**	11.129	0.013	0.194	0.115
**23**	2.954	0.001	1.221	0.784
**24**	11.161	0.092	0.479	0.055
**25**	11.418	75.898	0.068	0.138
**26**	1.975	120.449	0.000	0.227
**27**	15.958	0.000	1.568	0.132
**28**	2.930	0.000	0.000	0.543
**29**	2.846	0.000	0.219	0.307
**30**	1.883	0.000	0.939	0.466
**31**	9.085	0.000	1.766	0.253
**32**	5.280	0.000	0.000	0.190
**33**	3.886	0.000	0.105	0.188
**34**	8.090	0.005	0.000	0.303
**35**	3.715	0.000	0.403	0.267
**36**	0.776	17.815	0.000	0.161
**37**	10.299	0.002	0.010	0.111
**38**	5.102	0.000	0.226	0.152
**39**	3.872	0.000	0.015	0.287
**40**	2.856	0.000	0.264	0.265

b

Factor	U Loadings	Annotation
**23**	0.7839	Microglia/Macrophage
**28**	0.5429	Multiple Clusters
**14**	0.4933	Polydendrocytes
**1**	0.4902	Endothelial Stalk
**30**	0.4664	Mural
**13**	0.4607	Fibroblasts
**15**	0.3605	Endothelial Stalk
**6**	0.3472	Macrophage
**29**	0.3072	Noise
**34**	0.3034	Astrocytes

Supplementary Fig. 3. Relative contributions of metagenes to cell-type-specific reconstructions. Each matrix (W, V^1, V^2, and U) has a distinct contribution to the final matrix reconstruction (a), with the shared features, W, generally contributing the most, and the dataset-specific metagenes (V^1, V^2) contributing the least. The highest ten factor loadings, and their associated cell type, are shown for U matrix decomposition (b). The associated cell types are primarily non-neuronal.

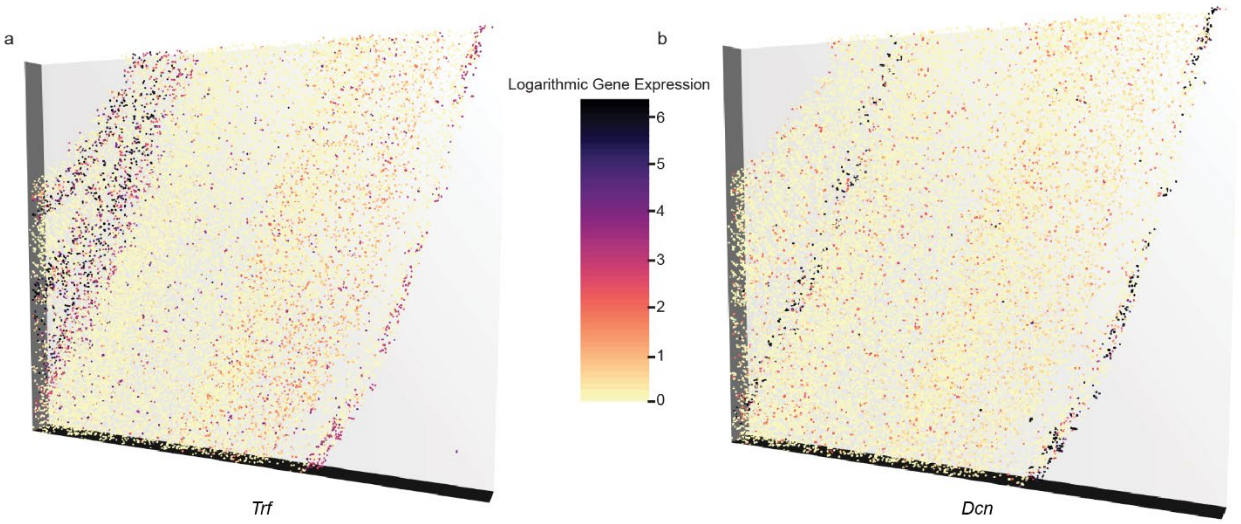

Supplementary Fig. 4. Spatial expression patterns imputed for *Trf* and *Dcn*. After performing KNN imputation, we are able to visualize the imputed expression profiles of the *Trf* (a) and *Dcn* (b) genes. *Trf*, a recognized marker gene of oligodendrocyte cells[32], consequently shows the greatest expression in the region composed of white matter. *Dcn* has previously been found in three types of vascular leptomeningeal cells (VLMCs)[33], which are known to comprise vascular structures. Therefore, we expected to see cells with highest imputed expression of *Dcn* expression near the blood brain barrier.

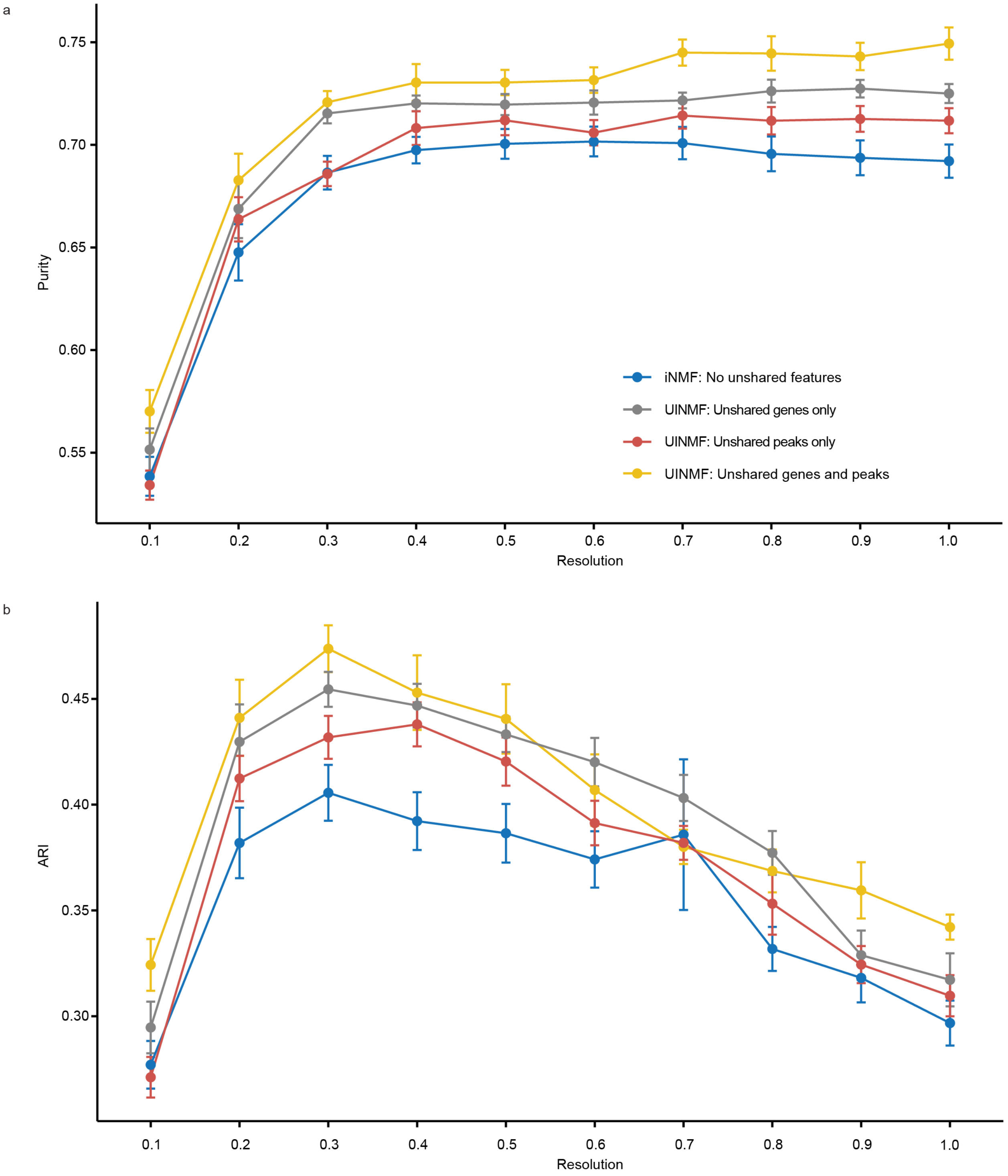

Supplementary Figure 5. Unshared genes and unshared chromatin peaks each improve integration performance. To quantify the individual benefit of using the unshared genes and unshared peaks, we perform the SNARE-seq and STARmap integration with no unshared features, only the unshared genes as unshared features, only the unshared chromatin peaks as unshared features, and both unshared genes and unshared chromatin regions as unshared features. To demonstrate how the inclusion of each set of features changes the integration

quality, we show the difference in purity (a) and ARI (b) metrics. Data are presented as mean values +/- SEM.

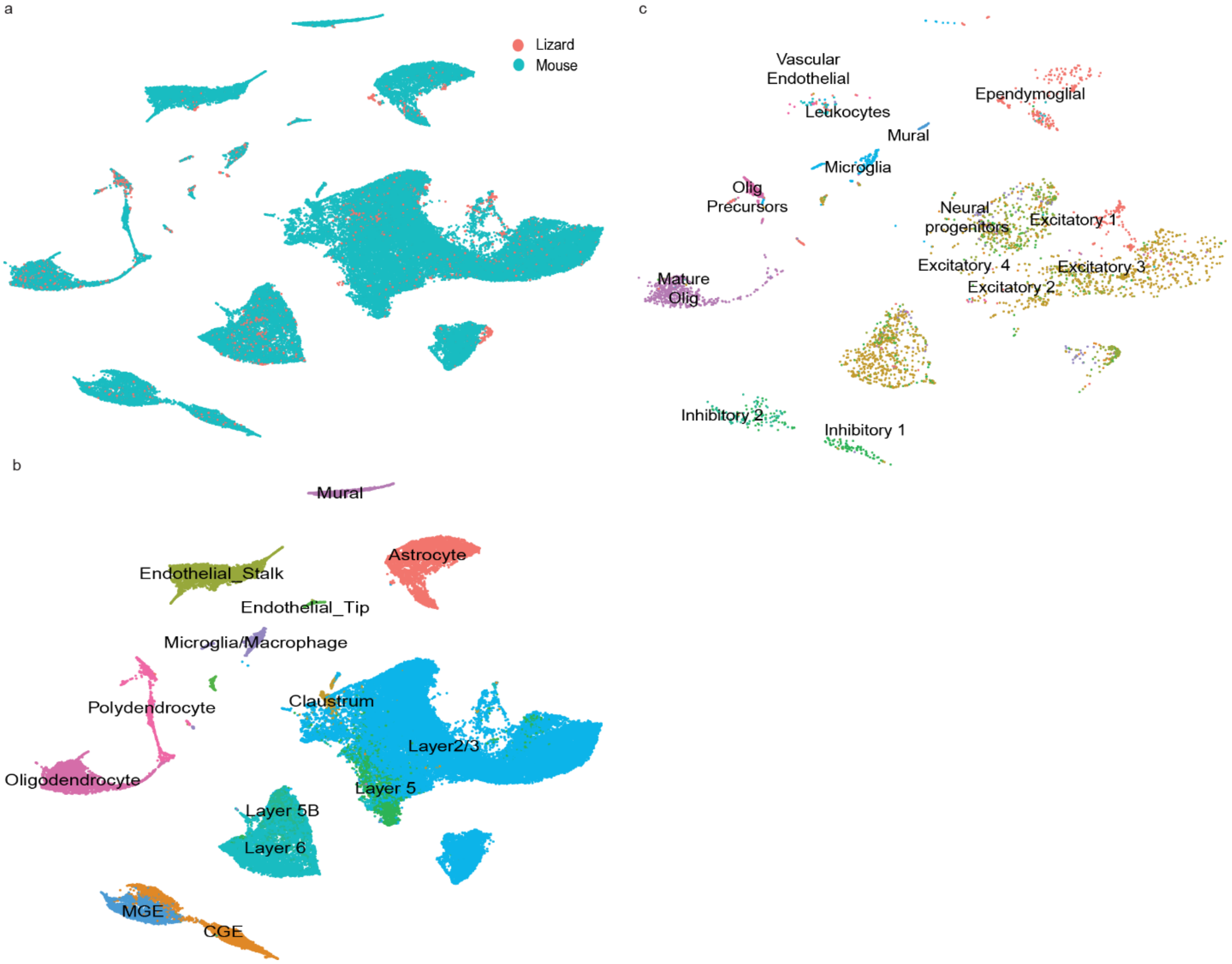

Supplementary Figure 6. Cross-Species Analysis using only Orthologous Genes. Integrating the lizard and mouse datasets using only orthologous genes, we still show adequate alignment between the two datasets (a). To examine cell type correspondence between the two datasets, we examined the mouse (b) and lizard (c) cells separately, labeled with their originally published labels.

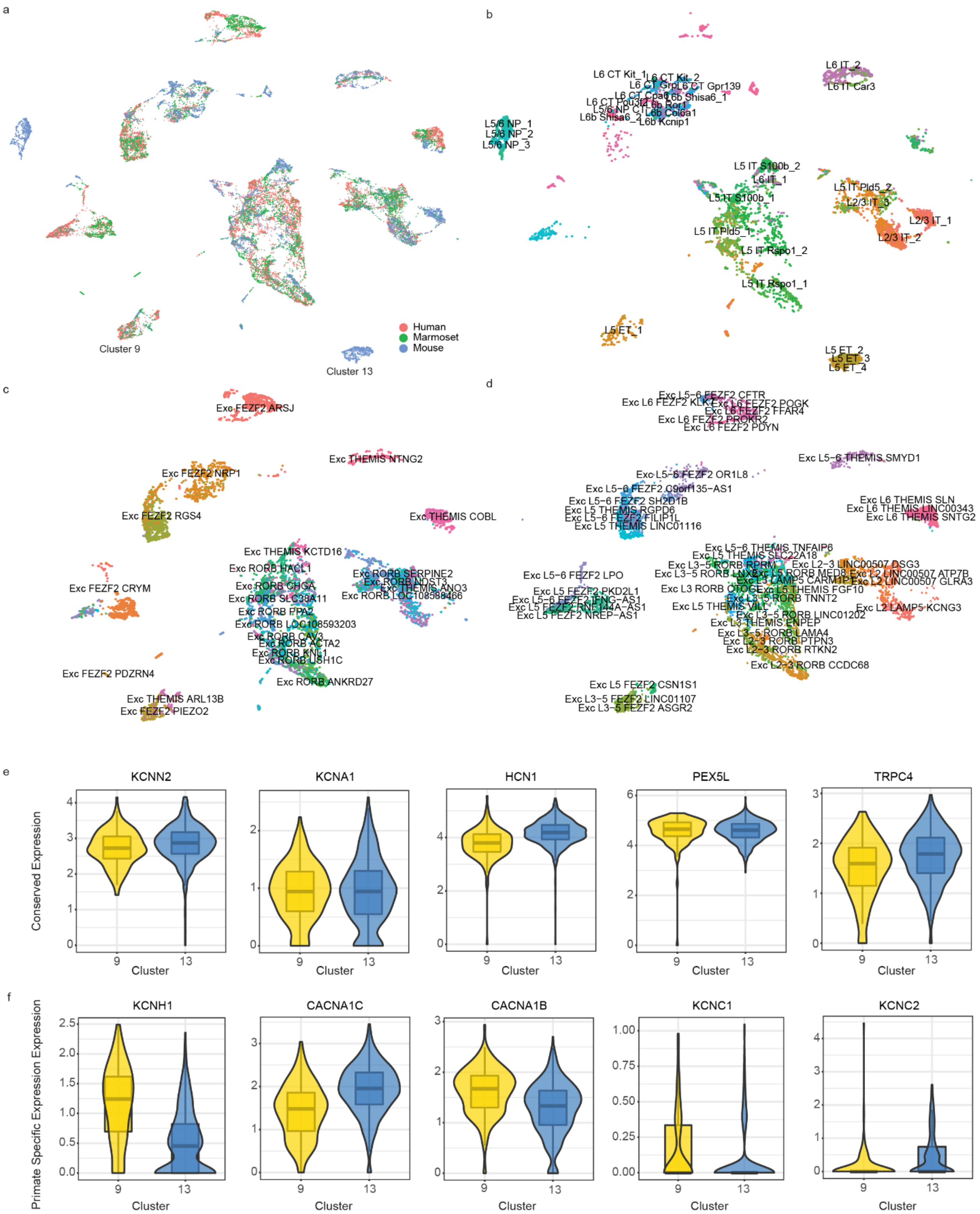

Supplementary Figure 7. UINMF integrates excitatory neurons from mouse, marmoset, and human using non-orthologous genes. After confirming that the cells from each species are generally well mixed within the latent space (a), we examine the correspondence between the originally published labels for the mouse (b), marmoset (c), and human (d). We further

investigate why only a single mouse L5 ET cluster aligns with the primate L5 ET neurons

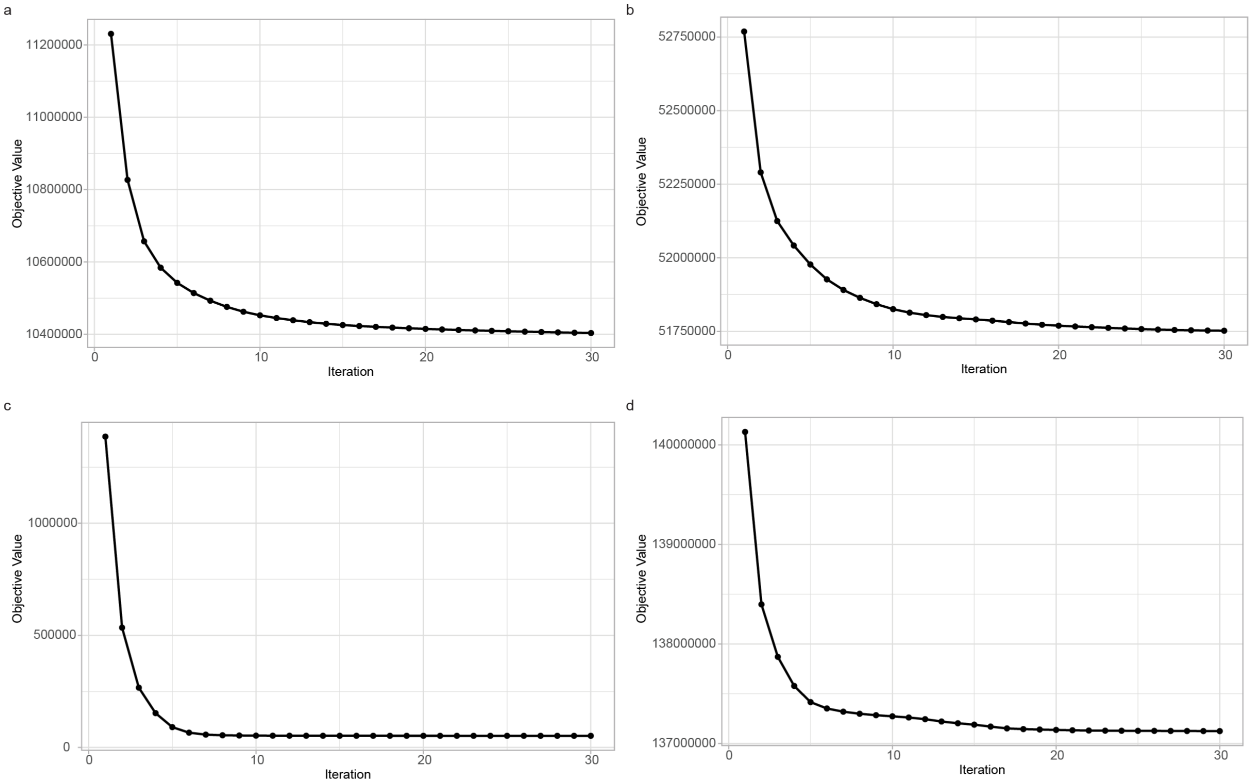

Supplementary Figure 8. BCD Algorithm for UINMF Converges Consistently. The objective function values for iNMF (a) and UINMF (b) across progressive iterations for the osmFISH and scRNA-seq analysis. The objective function values for iNMF (c) and UINMF (d) across progressive iterations for the SNARE-seq and STARmap data set integration. The first initialization is not shown, as it was large enough that including it resulted in the loss of the ability to visually identify the convergence of the successive objective values.

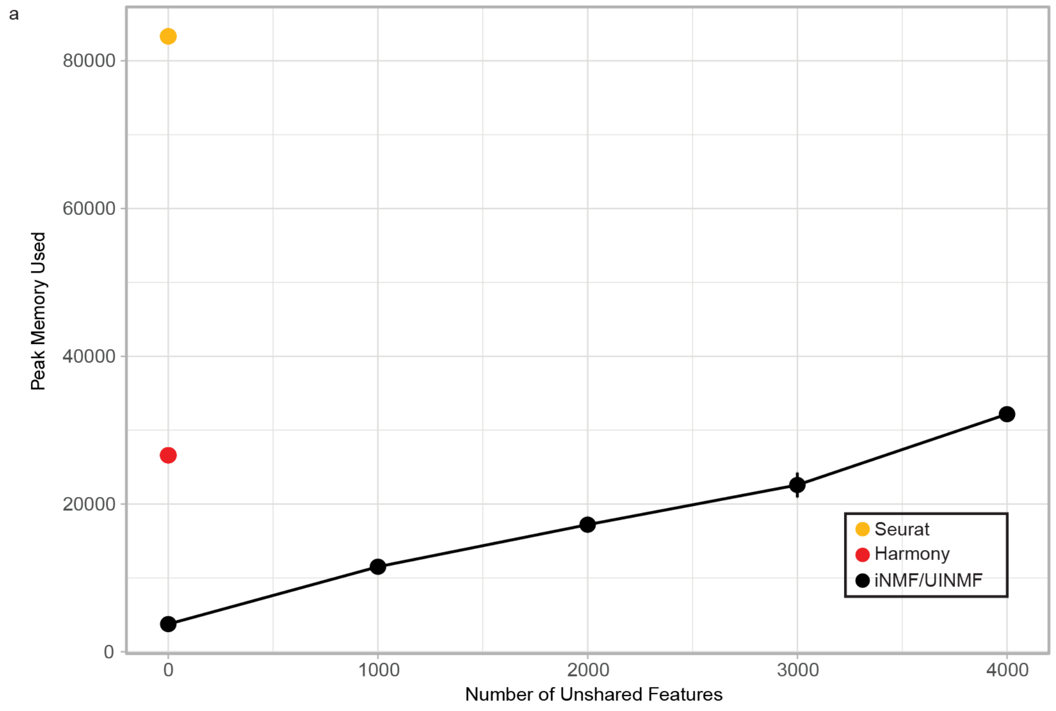

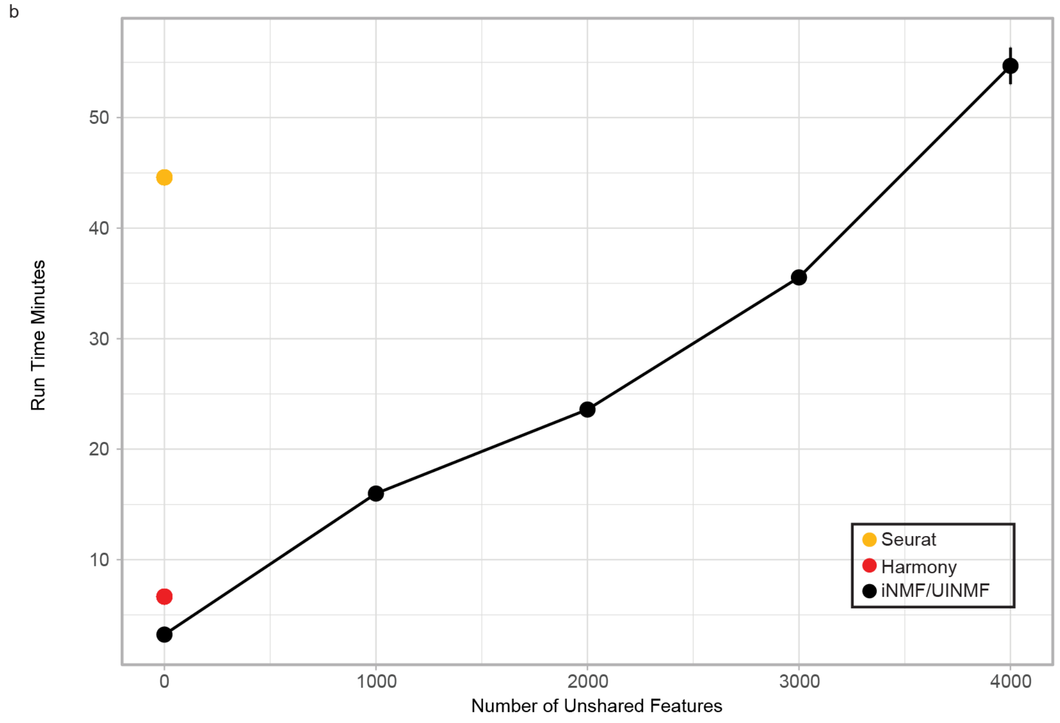

Supplementary Figure 9. The increase in time and memory usage resulting from the addition of extra features is manageable. We compare the difference in memory usage (a) and run time (b) over 5 initializations for each set of unshared features when integrating the STARmap data and scRNA-seq data (28 shared genes). Note that performing UINMF with zero additional features is the equivalent of performing iNMF. Data are presented as mean values +/- SEM.

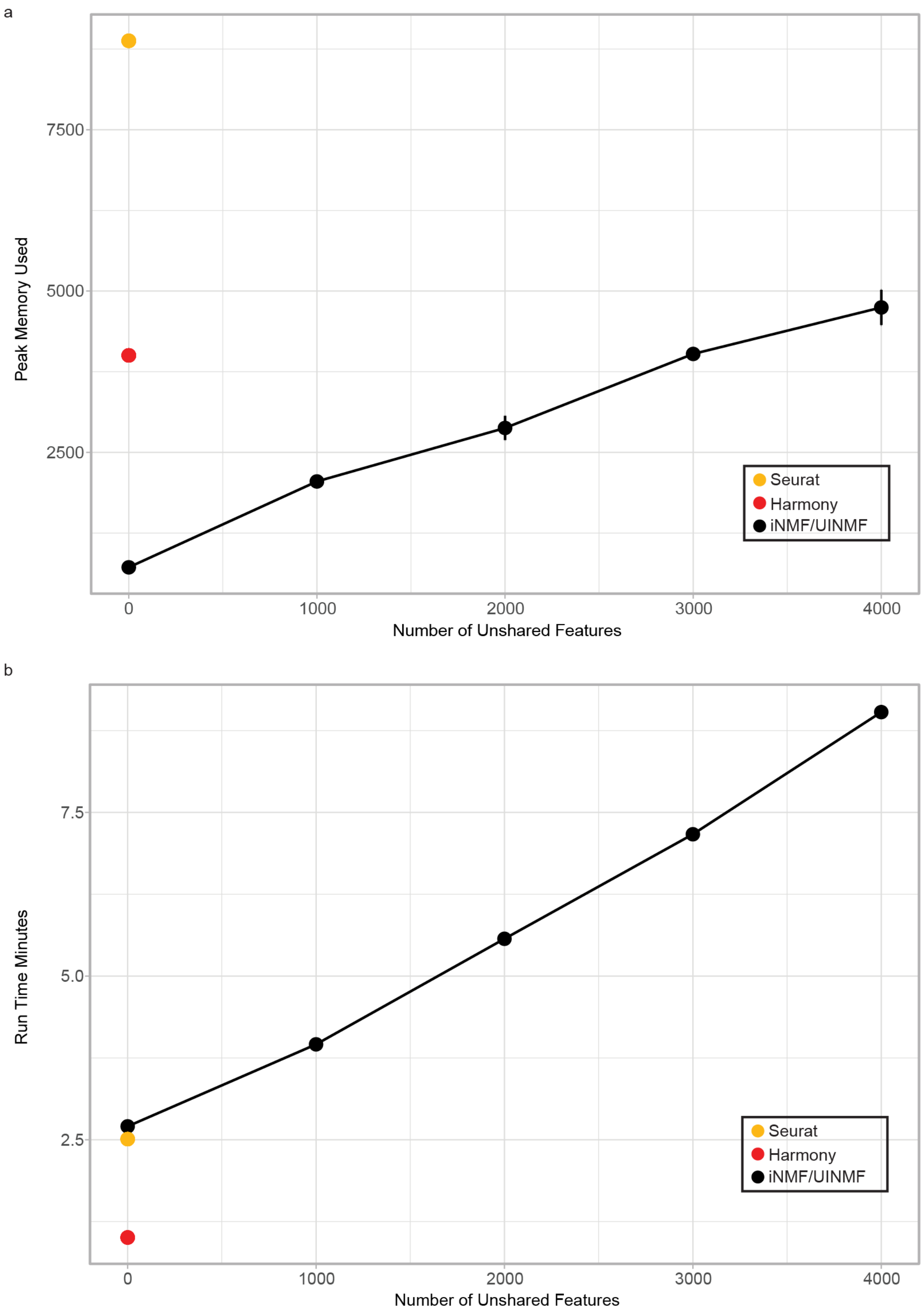

Supplementary Figure 10. The increased time and memory usage from using UINMF is appropriate for the increase in the number of features used. Using the SNARE-seq and STARmap data, we analyze the memory usage (a) and runtime (b) of UINMF, Seurat v3, and Harmony. There are 944 shared features. It should be noted that including 0 unshared features for UINMF is the equivalent of performing iNMF. Data are presented as mean values +/- SEM.

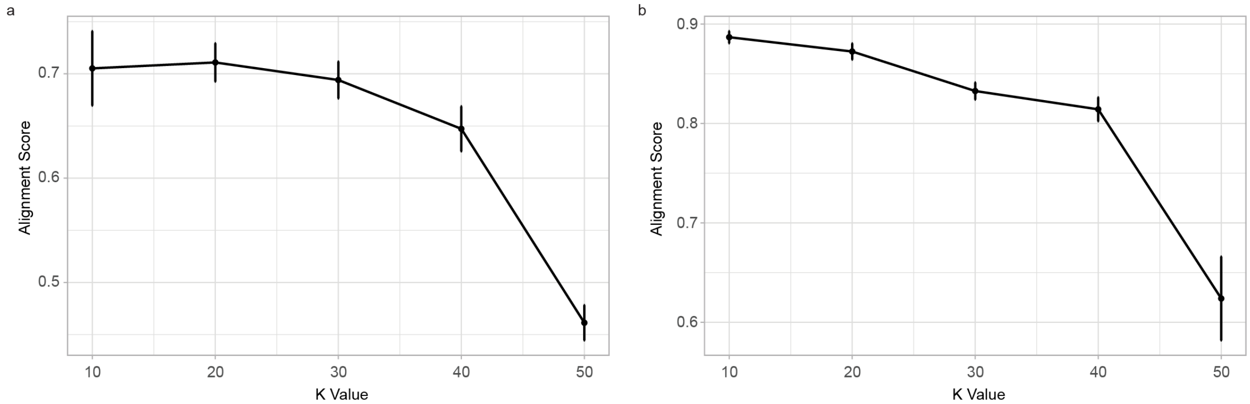

Supplementary Figure 11. UINMF Alignment Score Is Relatively Robust to K. The alignment scores for the STARMAP (a) and osmFISH (b) are not severely impacted by the choice of K until an unusually large value of K (K > 40) is chosen. Alignment scores were averaged over ten initializations. Data are presented as mean values +/- SEM.

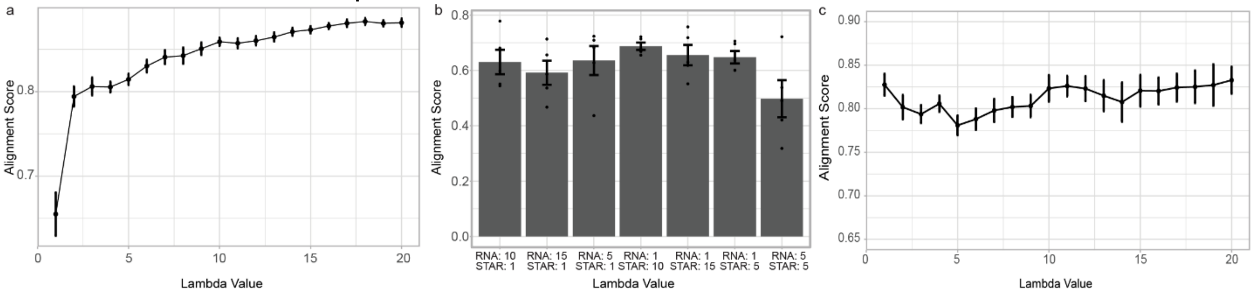

Supplementary Figure 12. UINMF Alignment Score Is Relatively Robust to Lambda. We calculated the alignment score of the algorithm across varying values of lambda for the SNARE-seq and STARmap data integration. The results of ten initializations are shown (a). For the STARmap data, we found the penalty 10,1, where the higher penalty is assessed to the STARmap dataset, yielded the highest alignment over 5 random initializations (b). Likewise, we calculated the alignment score over 10 random initializations for a variety of lambdas for the osmFISH integration (c). Data are presented as mean values +/- SEM.

Supplementary Table 1: Summary of shared and unshared features used for integration.

Dataset 1	Dataset 2	Dataset 1 Cells	Dataset 2 Cells	Shared Features	Unshared Features	Source of Unshared Features	Type of Unshared Features
SNARE-seq snATAC[2]	SNARE-seq scRNA[2]	10,309	10,309	2,589	2,000	Dataset 1	Intergenic Peaks
STARmap 3D[10]	DROPviz[29]	32,845	70,514	28	2,775	Dataset 2	Genes
osmFISH[12]	DROPviz[29]	6,471	70,020	33	2,000	Dataset 2	Genes
SNARE-seq[2]	STARmap 2D[10]	10,309	2,522	944	4,119	Dataset 1	Genes (2,668), Peaks (1,431)
Lizard[37]	DROPviz[29]	4,187	71,639	1,979	166	Dataset 1	Genes
