# Supplementary material for: UINMF performs mosaic integration of single-cell multi-omic datasets using nonnegative matrix factorization
Source: Nat Commun. 2022 Feb 9;13:780. doi: 10.1038/s41467-022-28431-4 (PMC8828882; doi:10.1038/s41467-022-28431-4)
Supplement: Supplementary file 2 — Reporting Summary [file 41467_2022_28431_MOESM2_ESM.pdf]

nature research

Corresponding author(s): Joshua D. Welch

Last updated by author(s): December 5, 2021

Reporting Summary

Nature Research wishes to improve the reproducibility of the work that we publish. This form provides structure for consistency and transparency in reporting. For further information on Nature Research policies, see our Editorial Policies and the Editorial Policy Checklist.

Statistics

For all statistical analyses, confirm that the following items are present in the figure legend, table legend, main text, or Methods section.

n/a	Confirmed	
☐	☒	The exact sample size (*n*) for each experimental group/condition, given as a discrete number and unit of measurement
☒	☐	A statement on whether measurements were taken from distinct samples or whether the same sample was measured repeatedly
☐	☒	The statistical test(s) used AND whether they are one- or two-sided<br>*Only common tests should be described solely by name; describe more complex techniques in the Methods section.*
☐	☒	A description of all covariates tested
☐	☒	A description of any assumptions or corrections, such as tests of normality and adjustment for multiple comparisons
☐	☒	A full description of the statistical parameters including central tendency (e.g. means) or other basic estimates (e.g. regression coefficient) AND variation (e.g. standard deviation) or associated estimates of uncertainty (e.g. confidence intervals)
☐	☒	For null hypothesis testing, the test statistic (e.g. *F*, *t*, *r*) with confidence intervals, effect sizes, degrees of freedom and *P* value noted<br>*Give P values as exact values whenever suitable.*
☒	☐	For Bayesian analysis, information on the choice of priors and Markov chain Monte Carlo settings
☒	☐	For hierarchical and complex designs, identification of the appropriate level for tests and full reporting of outcomes
☒	☐	Estimates of effect sizes (e.g. Cohen's *d*, Pearson's *r*), indicating how they were calculated

Our web collection on statistics for biologists contains articles on many of the points above.

Software and code

Policy information about availability of computer code

Data collection	No data collection was performed.
Data analysis	An R implementation of the UINMF algorithm is available as part of the LIGER package: https://github.com/welch-lab/liger.<br>Statistical Analysis was performed using R (4.0.0).

For manuscripts utilizing custom algorithms or software that are central to the research but not yet described in published literature, software must be made available to editors and reviewers. We strongly encourage code deposition in a community repository (e.g. GitHub). See the Nature Research guidelines for submitting code & software for further information.

Data

Policy information about availability of data

All manuscripts must include a data availability statement. This statement should provide the following information, where applicable:

- Accession codes, unique identifiers, or web links for publicly available datasets
- A list of figures that have associated raw data
- A description of any restrictions on data availability

All datasets used in this paper are previously published and freely available.

- Mouse Frontal Cortex SNARE-seq cells from Chen et al.(2) are in the GEO database under accession code GSE126074 [https://www.ncbi.nlm.nih.gov/geo/query/acc.cgi?acc=GSE126074]
- Mouse frontal cortex spatial transcriptomic cells from Moffit et al.(10)[https://www.starmapresources.org/data]
- Mouse cells from the somatosensory cortex (12) [http://linnarssonlab.org/osmFISH/]
- Adult mouse brain cells from Saunders et al. (29) [http://dropviz.org/]
- Lizard Pallium cells from Tosches, et. al.(37) [https://public.brain.mpg.de/Laurent/ReptilePallium2018/]

● Mouse Primary Motor Cortex (10X and SMARTseq datasets) from Yao et al.(13) [https://assets.nemoarchive.org/dat-ch1nqb7]
● Mouse, Marmoset, and Human Primary Motor Cortex from Bakken et al.(38) [http://data.nemoarchive.org/publication_release/Lein_2020_M1_study_analysis/Transcriptomics/sncell/10X/]

Field-specific reporting

Please select the one below that is the best fit for your research. If you are not sure, read the appropriate sections before making your selection.

- [x] Life sciences
- [ ] Behavioural & social sciences
- [ ] Ecological, evolutionary & environmental sciences

For a reference copy of the document with all sections, see nature.com/documents/nr-reporting-summary-flat.pdf

Life sciences study design

All studies must disclose on these points even when the disclosure is negative.

Sample size	The sample size in this study is the number of cells included in the single-cell datasets we analyzed. Because we were using previously published, existing datasets, we did not have any control over the sample size (it was chosen by the experimental biologists who previously published the data).
Data exclusions	No data was excluded, but some cells were filtered during the preprocessing stage of our analysis. Criteria for filtering was provided by biological context (we filtered the STARmap data for hippocampal and caudal cells, as these were not the target of the original assay) or the original publication's quality control analysis (we filtered cells flagged as low-quality in the original study). We also removed a few cells that did not express any of the shared genes used for integration (because these had no data that could be used for integration). The details of each of these cases are described in the Methods section.
Replication	Because we were using previously published, existing datasets, we did not have any control over replication. All datasets that we used are publicly available, and we do not have any way to replicate them since we cannot perform experiments.
Randomization	Our study involved no patients or model organisms. We also did not apply any treatments or conditions; therefore, randomization is not relevant.
Blinding	Our study involved no patients or model organisms. We also did not apply any treatments or conditions; therefore, blinding is not relevant.

Reporting for specific materials, systems and methods

We require information from authors about some types of materials, experimental systems and methods used in many studies. Here, indicate whether each material, system or method listed is relevant to your study. If you are not sure if a list item applies to your research, read the appropriate section before selecting a response.

Materials & experimental systems

n/a	Involved in the study	
x		Antibodies
x		Eukaryotic cell lines
x		Palaeontology and archaeology
x		Animals and other organisms
x		Human research participants
x		Clinical data
x		Dual use research of concern

Methods

n/a	Involved in the study	
x		ChIP-seq
x		Flow cytometry
x		MRI-based neuroimaging
